# Fibroblast expression of transmembrane protein smoothened governs microenvironment characteristics after acute kidney injury

**DOI:** 10.1172/JCI165836

**Published:** 2024-05-07

**Authors:** Yuan Gui, Haiyan Fu, Zachary Palanza, Jianling Tao, Yi-Han Lin, Wenjian Min, Yi Qiao, Christopher Bonin, Geneva Hargis, Yuanyuan Wang, Peng Yang, Donald L. Kreutzer, Yanlin Wang, Yansheng Liu, Yanbao Yu, Youhua Liu, Dong Zhou

**Affiliations:** 1 Division of Nephrology, Department of Medicine, University of Connecticut School of Medicine, Farmington, Connecticut, USA.; 2 Department of Pathology, University of Pittsburgh School of Medicine, Pittsburgh, Pennsylvania, USA.; 3 Division of Nephrology, Department of Medicine, Stanford University School of Medicine, Stanford, California, USA.; 4 National Center for Advancing Translational Sciences, Rockville, Maryland, USA.; 5 State Key Laboratory of Natural Medicines and Jiangsu Key Laboratory of Drug Design and Optimization, China Pharmaceutical University, Nanjing, China.; 6 Department of Surgery and; 7 University of Connecticut, School of Medicine, Farmington, Connecticut, USA.; 8 Yale Cancer Biology Institute, Yale University, West Haven, Connecticut, USA.; 9 Department of Pharmacology, School of Medicine, Yale University, New Haven, Connecticut, USA.; 10 Department of Chemistry & Biochemistry, University of Delaware, Newark, Delaware, USA.

**Keywords:** Nephrology, Extracellular matrix, Proteomics

## Abstract

The smoothened (Smo) receptor facilitates hedgehog signaling between kidney fibroblasts and tubules during acute kidney injury (AKI). Tubule-derived hedgehog is protective in AKI, but the role of fibroblast-selective Smo is unclear. Here, we report that Smo-specific ablation in fibroblasts reduced tubular cell apoptosis and inflammation, enhanced perivascular mesenchymal cell activities, and preserved kidney function after AKI. Global proteomics of these kidneys identified extracellular matrix proteins, and nidogen-1 glycoprotein in particular, as key response markers to AKI. Intriguingly, Smo was bound to nidogen-1 in cells, suggesting that loss of Smo could affect nidogen-1 accessibility. Phosphoproteomics revealed that the ‘AKI protector’ Wnt signaling pathway was activated in these kidneys. Mechanistically, nidogen-1 interacted with integrin β1 to induce Wnt in tubules to mitigate AKI. Altogether, our results support that fibroblast-selective Smo dictates AKI fate through cell-matrix interactions, including nidogen-1, and offers a robust resource and path to further dissect AKI pathogenesis.

## Abstract 2

Fibroblast-derived smoothened interacts with nidogen-1 to create a favorable microenvironment that protects against acute kidney injury.

## Graphical abstract

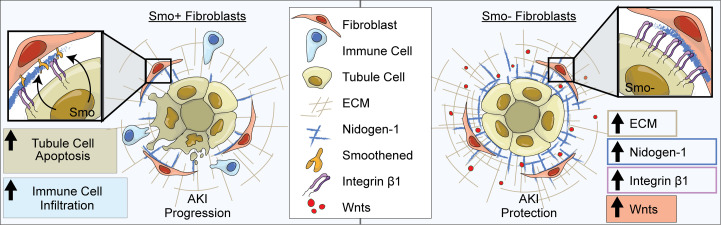

## Introduction

Acute kidney injury (AKI) is a clinical syndrome characterized by an abrupt decline in renal function within a few hours or days, which is associated with high morbidity and mortality globally (1). Although developing effective treatments for human AKI remains a challenge, advances in research have widened our knowledge of the pathogenesis of this refractory disease. In AKI induced by ischemia, sepsis, nephrotoxins, or major surgery, renal tubules have been the well-acknowledged epicenter of damage (2). However, emerging evidence shows that resident perivascular mesenchymal cells, such as fibroblasts and pericytes, are instinctively mobilized to participate in the repair process once AKI occurs (3–6). Unfortunately, the role of fibroblasts in AKI remains a mystery.

Fibroblasts are becoming increasingly appreciated for their association with AKI prognosis (7–9). Studies have demonstrated that after AKI, fibroblast activation precedes tubular cell proliferation (4). Early activated fibroblasts immediately release signals to neighboring cells, such as tubular epithelial or other perivascular mesenchymal cells, to rescue kidneys from AKI through auto- or paracrine mechanisms. Several highly conserved cell signaling systems are recruited for the acute phase of kidney injury/repair, including Hedgehog (Hh), Wnt, Notch, and mammalian target of rapamycin (MTOR) (3, 10–16). Moreover, fibroblasts are the principal effector cells responsible for synthesizing the extracellular matrix (ECM) (17). After AKI, appropriate types and amounts of ECM are beneficial for forming a favorable microenvironment in the diseased kidneys (18). This microenvironment functionally serves as a physical home to allow injured cells to rest and as a scaffold backbone for tissue remodeling. Therefore, it is conceivable to speculate that fibroblasts have a unique role in regulating AKI recovery.

Smoothened (Smo) is a 7-pass transmembrane receptor that is a crucial positive regulator in the Hh signaling pathway. The Hh pathway is a highly evolutionarily conserved system linked to cell-cell communications for embryonic development and adult tissue homeostasis (19, 20). Its activity is triggered by stoichiometric binding of Hh ligands to Patched (Ptc) (21). Ptc itself is an inhibitory transmembrane receptor that keeps the Hh pathway off by inhibiting Smo in the absence of Hh (22). Once Hh is presented to Ptc, Smo is activated, which turns on downstream genes such as the glioma-associated oncogene homolog (Gli) transcription factor. Mammals have 3 Hh homologues, Desert (Dhh), Indian (Ihh), and Sonic (Shh), of which Shh is the best studied (23, 24). In the kidney, Shh is exclusively produced by tubules (4, 25), but fibroblasts are its specific target (26). In these responsive fibroblasts, how Smo regulates AKI remains unclear. Besides transducing signals, we ask whether fibroblast-selective Smo directly affects kidney protection by binding small molecules or large proteins to adjust cell behaviors and even the tissue microenvironment after AKI. Here, integrating traditional molecular pathology with systems biology, we illustrate how fibroblast-selective Smo controls kidney fate after AKI. From the perspective of cell-matrix-cell interactions, our study provides open-access data resources as a complementary piece of the puzzle for understanding AKI pathogenesis.

## Results

### Smo ablation in fibroblasts mitigates ischemic AKI.

**Figure 1:**
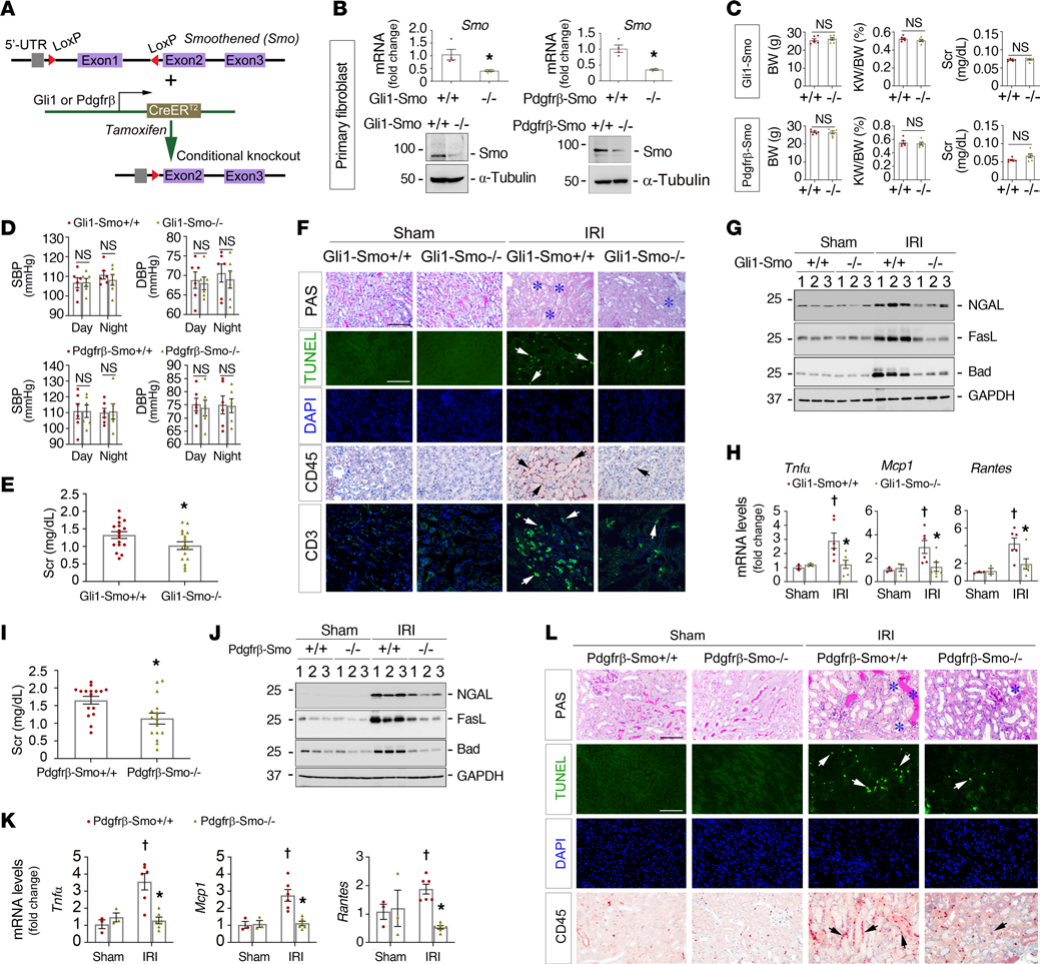
Fibroblast-specific ablation of Smo mitigates ischemic AKI. (**A**) Generation of Smo fibroblast-specific deletion mice. (**B**) Quantitative real-time PCR (qPCR) analysis (*n* = 4) and Western blot assay showing Smo levels in primary fibroblasts isolated from *Gli1-Smo^+/+^* and *Gli1-Smo^–/–^* or *Pdgfr*-β*-Smo^+/+^* and *Pdgfr*-β*-Smo^–/–^* kidneys. (**C**) The mouse baseline body weight (BW), kidney-to-body weight (KW/BW) ratios, serum creatinine (Scr), and (**D**) blood pressure levels (*n* = 6). At 1 day after IRI, (**E**) Scr levels in *Gli1-Smo^+/+^* and *Gli1-Smo^–/–^* mice (*n* = 16-17). (**F**) Periodic Acid–Schiff (PAS) staining showing kidney morphological changes in *Gli1-Smo^+/+^* and *Gli1-Smo^–/–^* mice. Blue asterisks indicate injured tubules. Representative micrographs of TUNEL or IHC staining against CD45 and CD3 in *Gli1-Smo^+/+^* and *Gli1-Smo^–/–^* kidneys. Scale bar: 25 μm. (**G**) Western blots assay of NGAL, FasL, and Bad in *Gli1-Smo^+/+^* and *Gli1-Smo^–/–^* kidneys (Sham, *n* = 3; IRI, *n* = 5). (**H**) qPCR analysis showing *Tnf-*α, *Mcp1*, and *Rantes* mRNA levels in *Gli1-Smo^+/+^* and *Gli1-Smo^–/–^* kidneys (Sham, *n* = 3; IRI, *n* = 6). (**I**) Scr levels in *Pdgfr*-β*-Smo^+/+^* and *Pdgfr*-β*-Smo^–/–^* mice (*n* = 15-16). (**J**) Western blots showing NGAL, FasL, and Bad levels in *Pdgfr*-β*-Smo^+/+^* and *Pdgfr*-β*-Smo^–/–^* kidneys. (**K**) qPCR analysis showing *Tnf-*α, *Mcp1*, and *Rantes* mRNA levels in *Pdgfr*-β*-Smo^+/+^* and *Pdgfr*-β*-Smo^–/–^* kidneys (Sham, *n* = 3; IRI, *n* = 6). (**L**) Representative micrographs of PAS, TUNEL, and IHC staining against CD45 in *Pdgfr*-β*-Smo^+/+^* and *Pdgfr*-β*-Smo^–/–^* kidneys. Scale bar: 25 μm. Arrows indicate positive cells. DAPI is a nuclear counterstain. For all Western blot panels, numbers indicate individual animals in a given group. †*P* < 0.05 versus sham control, *P < 0.05 versus *Gli1-Smo^+/+^* or *Pdgfr*-β*-Smo^+/+^* IRI mice. Dots indicate individual animals in a given group. Graphs are presented as means ± SEM. Differences among groups were analyzed using unpaired *t* tests or 1-way ANOVA followed by the Student-Newman-Keuls test.

To elucidate the role of fibroblast-selective *Smo* in AKI, we generated 2 strains of fibroblast-specific *Smo*-KO mice by employing the tamoxifen-inducible Cre-LoxP system (11). Briefly, as depicted in Figure 1A, homozygous *Smo*-floxed mice were mated with the *Gli1*-CreER^T2^ or platelet-derived growth factor receptor (*Pdgfr*) β-P2A-CreER^T2^ transgenic mice under the control of endogenous *Gli1* or *Pdgfr*-β promoter to create *Smo* fibroblast conditional-KO mice (genotype: Cre^+/–^, *Smo*^fl/fl^; designated as *Gli1-Smo^–/–^* or *Pdgfr*-β*-Smo^–/–^*); *Gli1* or *Pdgfr*-β expression is confined to a subset of fibroblasts (Figure 1A) (3, 11, 27–29). Age and sex-matched *Smo*-floxed mice (genotype: Cre^–/–^, *Smo*^fl/fl^; designated as *Gli1-Smo^+/+^* or *Pdgfr*-β*-Smo^+/+^*) from the same litters were used as controls (Supplemental Figure 1, A and B; supplemental material available online with this article; https://doi.org/10.1172/JCI165836DS1)). *Gli1-Smo^–/–^* and *Pdgfr*-β*-Smo^–/–^* mice were phenotypically normal. After tamoxifen injections for 5 consecutive days, Cre-mediated recombination was induced in *Gli1^+^* or *Pdgfr*-β*^+^* fibroblasts, as previously reported (11, 30–32). To confirm the knockout efficiency, we further isolated primary kidney fibroblasts from the above mice (approximately 10 days old) and deleted *Smo* using hydroxytamoxifen. Quantitative real-time PCR (qPCR) and Western blot analyses demonstrated marked reductions of Smo in the isolated fibroblasts from *Gli1-Smo^–/–^* and *Pdgfr*-β*-Smo^–/–^* mice (Figure 1B). There was no appreciable abnormality and difference in body weight, kidney-to-body weight ratios, serum creatinine, and blood pressures between *Gli1-Smo^+/+^* and *Gli1-Smo^–/–^* or *Pdgfr*-β*-Smo^+/+^* and *Pdgfr*-β*-Smo^–/–^* mice (Figure 1, C and D). Then, after 1-week of tamoxifen wash out, the mice were subjected to 30 minutes of bilateral renal ischemia-reperfusion injury (IRI) to induce AKI and allowed to recover for a day. Surprisingly, in contrast with deletion of Shh in tubules (4), deletion of *Smo* in Gli1^+^ fibroblasts preserved kidney function at 1 day after ischemic AKI. Compared with *Gli1-Smo^+/+^* mice subjected to ischemic AKI, serum creatinine levels were reduced by 22.7% in *Gli1-Smo^–/–^* mice following ischemic AKI (Figure 1E). Consistently, *Gli1-Smo^–/–^* mice exhibited reduced AKI-associated morphologic changes, such as less cellular debris, brush border loss, and intratubular proteinaceous casts (Figure 1F; Quantitative data shown in Supplemental Figure 2A). Further, the expression of the classic acute tubular injury marker, neutrophil gelatinase-associated lipocalin (NGAL) (33), was also decreased in *Gli1-Smo^–/–^* mice at 1 day after ischemic AKI, compared with *Gli1-Smo^+/+^* mice (Figure 1G; Quantitative data shown in Supplemental Figure 2B).

To confirm the above phenotypic changes, we assessed the differences in the key biological mechanisms involved in AKI development — inflammation and cell death (34, 35) — between *Gli1-Smo^+/+^* and *Gli1-Smo^–/–^* ischemic kidneys in mice. qPCR analysis revealed that tumor necrosis factor–α (*Tnf-*α), monocyte chemoattractant protein-1 (*Mcp1*), and regulated on activation, normal T cell expressed and secreted (*Rantes*) mRNA levels were markedly reduced in *Gli1-Smo^–/–^* mice compared with *Gli1-Smo^+/+^* mice (Figure 1H). Accordingly, immunostaining results indicated fewer infiltrated CD45^+^ monocytes and CD3^+^ T cells in diseased kidneys of *Gli1-Smo^–/–^* mice (Figure 1F; Quantitative data in Supplemental Figure 2, C and D).

Because AKI features sublethal or lethal damage of kidney tubules (35), we further examined if loss of *Smo* in *Gli1*^+^ fibroblasts influence different types of tubular cell death in ischemic kidneys, such as apoptosis, necrosis, or ferroptosis. Western blots revealed that proapoptotic proteins FasL and Bad were reduced in *Gli1-Smo^–/–^* mice compared with *Gli1-Smo^+/+^* mice (Figure 1G; Quantitative data shown in Supplemental Figure 2B). Terminal deoxynucleotidyl transferase dUTP nick end labeling (TUNEL) showed similar results (Figure 1F; Quantitative data in Supplemental Figure 2E). Intriguingly, neither Glutathione Peroxidase 4 (GPX4) nor phosphorylated mixed lineage kinase domain-like protein (pMLKL) was changed between *Gli1-Smo^+/+^* and *Gli1-Smo^–/–^* kidneys after ischemic AKI (Supplemental Figure 2, F and G).

Similar to the findings in *Gli1-Smo^–/–^* mice, after IRI at 1 day, *Pdgfr*-β*-Smo^–/–^* mice also exhibited preserved serum creatinine levels (31.4% reduction, Figure 1I), reduced protein expression of NGAL, FasL, and Bad (Figure 1J), and improved secretion of chemocytokines (Figure 1K) compared with *Pdgfr*-β*-Smo^+/+^* mice. PAS staining, TUNEL assay, and IHC staining against CD45 consistently confirmed the results (Figure 1L). The quantitative data is presented in Supplemental Figure 2, H–K. Collectively, these results suggested that ablation of *Smo* in *Gli1*^+^ or *Pdgfr*-β^+^ fibroblasts protect against ischemic AKI.

### Loss of Smo in fibroblasts enhance perivascular mesenchymal cell activity after AKI.

**Figure 2:**
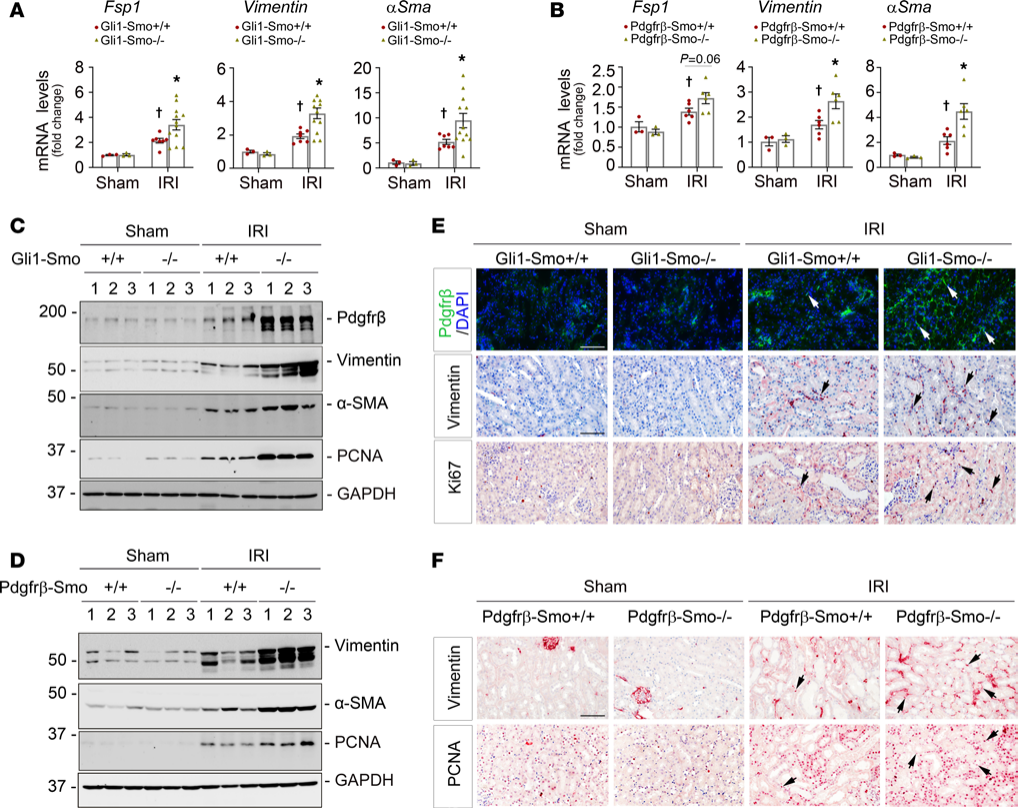
Fibroblast-specific ablation of Smo promotes perivascular mesenchymal cell activation and proliferation after AKI. At 1 day after IRI, (**A**) qPCR analyses of *Fsp1*, *vimentin*, and α*Sma* mRNA in *Gli1-Smo^+/+^* and *Gli1-Smo^–/–^* kidneys (Sham, *n* = 3; IRI, *n* = 7-12). (**B**) qPCR analyses of *Fsp1*, *vimentin*, and α*Sma* mRNA in *Pdgfr*-β*-Smo^+/+^* and *Pdgfr*-β*-Smo^–/–^* kidneys (Sham, *n* = 3; IRI, *n* = 6). (**C**) Western blot assay of Pdgfr-β, vimentin, αSMA, and PCNA proteins in *Gli1-Smo^+/+^* and *Gli1-Smo^–/–^* kidneys. (**D**) Western blots assay of vimentin, αSMA, and PCNA proteins in *Pdgfr*-β*-Smo^+/+^* and *Pdgfr*-β*-Smo^–/–^* kidneys. (**E**) Representative micrographs of Pdgfr-β, vimentin, and Ki67 expression in *Gli1-Smo^+/+^* and *Gli1-Smo^–/–^* kidneys. Arrows indicate positive cells. Scale bar: 25 μm. (**F**) Representative micrographs of vimentin and PCNA expression in *Pdgfr*-β*-Smo^+/+^* and *Pdgfr*-β*-Smo^–/–^* kidneys. Scale bar: 25 μm. Arrows indicate positive cells. DAPI is a nuclear counterstain. For all Western blot panels, numbers indicate individual animals in a given group. †*P* < 0.05 versus sham control, *P < 0.05 versus *Gli1-Smo^+/+^* or *Pdgfr*-β*-Smo^+/+^* IRI mice. Dots indicate individual animals in a given group. Graphs are presented as means ± SEM. Differences among groups were analyzed using 1-way ANOVA, followed by the Student-Newman-Keuls test.

Hh/Smo/Gli1 signaling is necessary for fibroblast proliferation in various diseases such as kidney, liver, lung, and heart fibrosis, systemic sclerosis, and cancer microenvironment formation (25, 36–42). We previously reported that early activated fibroblasts are indispensable for kidney repair after AKI (4). Therefore, we were puzzled as to why loss of fibroblast-selective *Smo* alleviated AKI in our current model. Considering that *Gli1*^+^ or *Pdgfr*-β^+^ fibroblasts are a small fraction of the perivascular mesenchymal cell population (11, 30), we speculated that loss of Smo in *Gli1*^+^ or *Pdgfr*-β^+^ fibroblasts may naturally activate the remaining perivascular mesenchymal cells as compensation, such as non-*Gli1*^+^ and non-*Pdgfr*-β^+^ fibroblasts/pericytes, to enhance cell survival after AKI. To this end, we systemically assessed the expression of several markers for activated fibroblasts/pericytes in diseased kidneys, including fibroblast-specific protein 1 (Fsp1), vimentin, α-smooth muscle actin (αSma), and Pdgfr-β. qPCR analyses revealed that mRNA expression of *Fsp1*, *vimentin*, and α*Sma* were substantially increased in *Gli1-Smo^–/–^* mice 1 day after IRI compared with *Gli1-Smo^+/+^* mice (Figure 2A). Consistently, *Pdgfr*-β*-Smo^–/–^* mice showed similar results after IRI (Figure 2B). Western blots further demonstrated marked elevation of Pdgfr-β, vimentin, or α-SMA proteins in *Gli1-Smo^–/–^* or *Pdgfr*-β*-Smo^–/–^* mice (Figure 2, C and D; quantitative data are presented in Supplemental Figure 3, A and C). Consistently, immunostaining revealed increased expression of Pdgfr-β or vimentin in diseased kidneys of *Gli1-Smo^–/–^* or *Pdgfr*-β*-Smo^–/–^* mice (Figure 2, E and F; quantitative data are presented in Supplemental Figure 3, B and D). To understand the distribution of proliferative cells in diseased kidneys after loss of Smo in *Gli1*^+^ or *Pdgfr*-β^+^ fibroblasts, we assessed proliferating cell nuclear antigen (PCNA) and Ki67 levels. Western blot assays demonstrated an upregulation of PCNA in *Gli1-Smo^–/–^* or *Pdgfr*-β*-Smo^–/–^* mice at 1 day after IRI compared with their corresponding littermate controls (Figure 2, C and D; quantitative data are presented in Supplemental Figure 3, A and C), and IHC staining further confirmed that the number of Ki67^+^ or PCNA^+^ interstitial cells were larger in *Gli1-Smo^–/–^* or *Pdgfr*-β*-Smo^–/–^* kidneys than in the kidneys of their littermate controls (Figure 2, E and F). Taken together, these results indicated that ablation of *Smo* in fibroblasts in turn activated perivascular mesenchymal cells after ischemic AKI.

### Global proteomics identifies nidogen-1 as a key participant in AKI repair after loss of Smo in fibroblasts.

**Figure 3:**
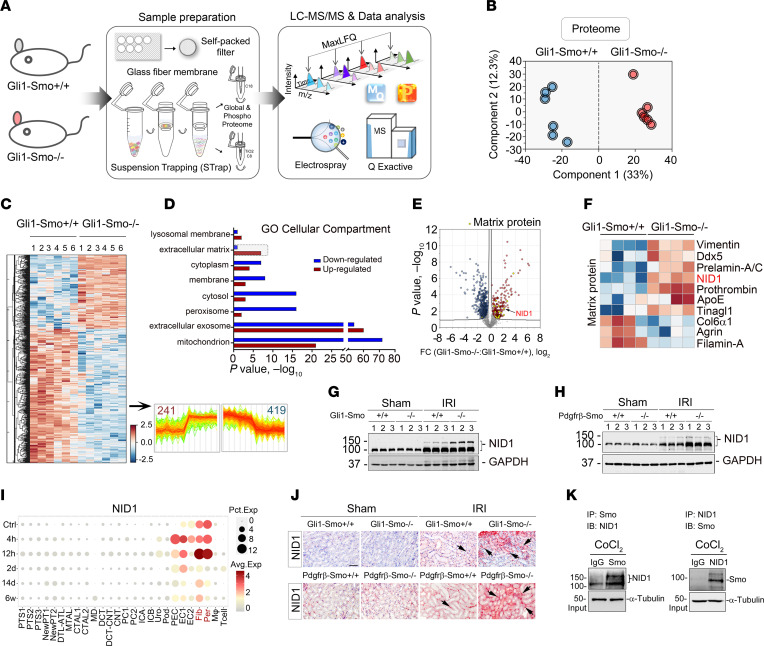
Global proteomics identifies NID1 as a prominent matrix protein in Gli1^+^ fibroblast-specific Smo deletion kidneys after AKI. (**A**) Experimental workflow of the global proteomic analysis. 6 mice in each group were used for mass spectrometry. (**B**) Principal component analysis of global proteomes from *Gli1-Smo^+/+^* and *Gli1-Smo^–/–^* kidneys 1 day after ischemic AKI. (**C**) Heatmap of ANOVA-significant proteins. Label-free quantitation (LFQ) intensity of represented proteins were z-scored and plotted according to the color bar. 2 clusters of proteins with different patterns of abundance profiles are highlighted in the heatmap. (**D**) GO cellular compartment terms in each cluster of proteins are plotted with their names and significance. ECM proteins are boxed to indicate the protein group with the largest difference in upregulated proteins. (**E**) Volcano plot shows the differential proteins between *Gli1-Smo^+/+^* and *Gli1-Smo^–/–^* kidneys. Up and down regulated proteins (FC, fold-change) are colored in red and blue, respectively. Yellow dots indicate ECM proteins. (**F**) Heatmap of differentially expressed ECM proteins. (**G** and **H**) Western blots of NID1 protein in *Gli1-Smo^+/+^* and *Gli1-Smo^–/–^* or in *Pdgfr*-β*-Smo^+/+^* and *Pdgfr*-β*-Smo^–/–^* kidneys 1 day after IRI. Numbers indicate individual animals in a given group. (**I**) Single nucleus RNA-Seq showing NID1 mainly expressed by fibroblasts (Fib) and pericytes (Per) 12 hours after IRI. (Data were extracted from the online database provided by Benjamin Humphrey’s laboratory, http://humphreyslab.com/SingleCell/displaycharts.php) (**J**) IHC staining showing NID1 protein expression in *Gli1-Smo^+/+^* and *Gli1-Smo^–/–^* or in *Pdgfr*-β*-Smo^+/+^* and *Pdgfr*-β*-Smo^–/–^* kidneys 1 day after IRI. Arrows indicate positive staining. Scale bar: 25 μm. (**K**) Immunoprecipitation revealing that Smo binds to NID1. NRK-49F cells under CoCl_2_ stress were immunoprecipitated with Smo or NID1 antibody, followed by immunoblotting with antibody against NID1 (left blot) or Smo (right blot). Differences among groups were analyzed using unpaired *t* tests.

To better understand how loss of *Smo* in fibroblasts mitigates AKI, we used a label-free quantitative approach to profile the global proteome of the kidneys of *Gli1-Smo^+/+^* and *Gli1-Smo^–/–^* mice at 1 day after IRI (Figure 3A). The principal component analysis (PCA) of our proteomic data clearly classified *Gli1-Smo^+/+^* and *Gli1-Smo^–/–^* mice according to their genotype (Figure 3B; quantified proteins are presented in Supplemental Table 1). A 2-tailed *t* test identified 660 differentially expressed proteins (Permutation FDR 0.05) between the diseased kidneys of *Gli1-Smo^+/+^* and *Gli1-Smo^–/–^* mice. Compared with *Gli1-Smo^+/+^* mice, 241 and 419 proteins were up and down regulated, respectively, in *Gli1-Smo^–/–^* mice (Figure 3C and Supplemental Table 2; correlations between biological replicates within the same group and the distribution of protein intensity are presented in Supplemental Figure 4, A and B). The following Gene Ontology (GO) cellular compartment analysis revealed that these proteins were generally distributed in mitochondria, extracellular exosome, peroxisome, cytosol, and ECM (Figure 3D). The activation of perivascular mesenchymal cells in *Gli1-Smo^–/–^* mice is linked to enhanced ECM synthesis in diseased kidneys (Figure 2). Indeed, unlike most proteins that showed down regulated trends in other cellular compartments, 110 out of 141 ECM proteins were upregulated in *Gli1-Smo^–/–^* mice compared with *Gli1-Smo^+/+^* mice (Figure 3D). This phenomenon indicates that ECM remodeling by enhanced protein synthesis may play a critical role in *Gli1-Smo^–/–^* kidney repair after IRI.

We therefore focused on the activated ECM proteins with significant upregulations in the kidneys of *Gli1-Smo^–/–^* mice. To select the most significantly impacted ECM protein for further investigation, we excluded those that have been well studied [such as vimentin (Figure 2) and prelamin-A/C]. Among the remaining proteins, we paid attention to nidogen-1 (NID1), one of the most prominent matrix proteins upregulated in *Gli1-Smo^–/–^* mice (Figure 3, E and F and Supplemental Figure 4C), which has been reported as an essential component of the basement membrane that plays a role in cell interactions with the ECM. Since ECM is identified as a major cellular compartment of distributed proteins, we hypothesized that ECM could organize a favorable kidney local microenvironment to repair kidneys after AKI. Thus, we chose NID1 for further study.

To confirm our findings, we assessed NID1 levels in *Gli1-Smo^+/+^* and *Gli1-Smo^–/–^* or *Pdgfr*-β*-Smo^+/+^* and *Pdgfr*-β*-Smo^–/–^* mice. After IRI at 1 day, NID1 protein was induced in diseased kidneys compared with sham controls. Western blot assays demonstrated a marked elevation of NID1 protein in *Gli1-Smo^–/–^* or *Pdgfr*-β*-Smo^–/–^* mice (Figure 3, G and H; quantitative data are presented in Supplemental Figure 4, D and E), compared with their corresponding controls. In a separate analysis using single nucleus RNA-Seq, NID1 was highly expressed in fibroblasts/pericytes at 12 hours after IRI (Figure 3I) (43). Consistently, IHC staining confirmed enhanced NID1 distribution in the interstitium of *Gli1-Smo^–/–^* or *Pdgfr*-β*-Smo^–/–^* ischemic kidneys (Figure 3J). To further establish the molecular link between NID1 and Smo, we identified multiple potential binding sites of NID1 and Smo in the optimal conformation at pose 51 through virtual protein-protein ZDOCK and RDOCK algorithms (Supplemental Figure 5). Immunoprecipitation of Smo or NID1 of Smo or NID1 pulled down both proteins, indicating that Smo physically binds to NID1, which indicated that Smo physically binds to NID1 (Figure 3K). In addition, to obtain a broader view of the dysregulated pathways after loss of Smo in fibroblasts, we constructed a signaling network based on the STRING database. The analysis with the highest confidence score (cutoff = 0.9) resulted in 413 nodes and 1,815 interactions. (Supplemental Figure 4F). The majority of interactions and nodes were associated with Wnt, Hh, and fatty acid oxidation pathways. Collectively, these data suggested that loss of Smo in fibroblasts resulted in upregulation of NID1, which may orchestrate a favorable matrix and metabolic microenvironment to mitigate AKI.

### Phosphoproteomics reveals that Smo deletion in fibroblasts remodels the Wnt signaling pathway.

**Figure 4:**
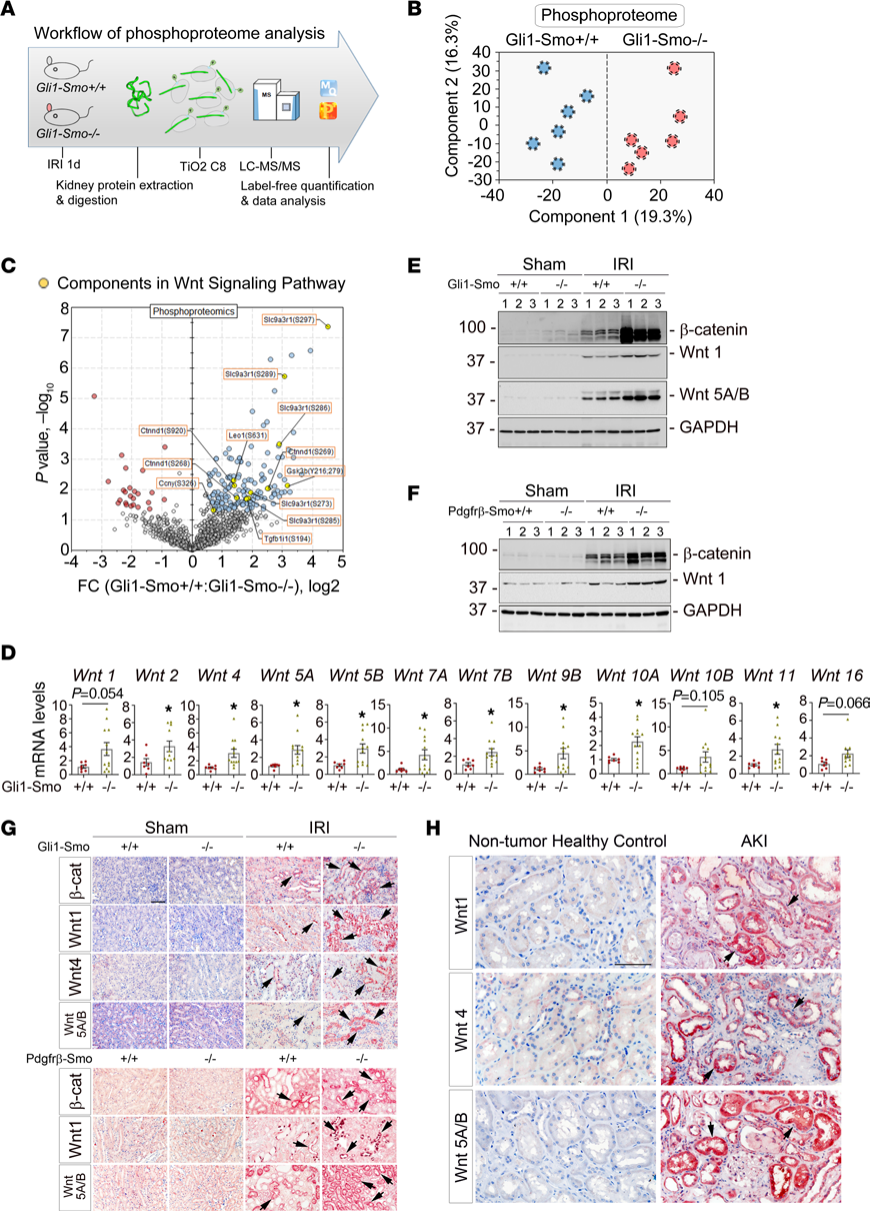
Phosphoproteomics reveals Wnt pathway activation in fibroblast-specific Smo-deletion kidneys after AKI. (**A**) Workflow of phosphoproteomics. (**B**) Principal component analysis of phosphoproteins from *Gli1-Smo^+/+^* and *Gli1-Smo^–/–^* kidneys 1 day after ischemic AKI. (**C**) Volcano plot of pairwise comparisons (fold-change, FC) between the kidney phosphoproteomes of *Gli1-Smo^+/+^* and *Gli1-Smo^–/–^* kidneys 1 day after ischemic AKI. (**D**) qPCR of *Wnt 1*, *2*, *4*, *5A*, *5B*, *7A*, *7B*, *9B*, *10A*, *10B*, *11*, and *16* mRNA in *Gli1-Smo^+/+^* and *Gli1-Smo^–/–^* kidneys 1 day after ischemic AKI. *P < 0.05 (*n* = 7–12). (**E**) Western blots of β-catenin, Wnt 1, and Wnt 5A/B proteins in *Gli1-Smo^+/+^* and *Gli1-Smo^–/–^* kidneys 1 day after ischemic AKI. Numbers indicate individual animals in a given group. (**F**) Western blots of β-catenin and Wnt 1 in *Pdgfr*-β*-Smo^+/+^* and *Pdgfr*-β*-Smo^–/–^* kidneys 1 day after ischemic AKI. Numbers indicate individual animals in a given group. (**G**) IHC staining showing β-catenin, Wnt1, Wnt 4, and Wnt5A/B in *Gli1-Smo^+/+^* and *Gli1-Smo^–/–^* or in *Pdgfr*-β*-Smo^+/+^* and *Pdgfr*-β*-Smo^–/–^* kidneys 1 day after ischemic AKI. Scale bar: 25 μm. (**H**) IHC staining showing Wnt1, Wnt 4, and Wnt5A/B expression in kidney biopsy specimens from patients with AKI. Arrows indicate positive staining. Scale bar: 25 μm. Graphs are presented as means ± SEM. Differences between groups were analyzed using unpaired *t* tests.

To gain more insights into the temporal regulation and functional changes in signaling of *Smo*-deficient fibroblasts in AKI repair, we performed phosphoproteomics on the same set of kidney samples used for the global proteomics (Figure 4A). Similar to the global proteomics, PCA of the phosphoproteome clearly separated the kidneys of *Gli1-Smo^+/+^* and *Gli1-Smo^–/–^* mice (Figure 4B). Impressively, most of the significantly different phosphopeptides were decreased in *Gli1-Smo^–/–^* mice compared with *Gli1-Smo^+/+^* mice (Supplemental Table 3). We then filtered the phosphorylation data set to include those phosphopeptides that were quantified in at least 3 replicates and used t-test analysis with *P* value correction (Permutation FDR 0.05). Enrichment analysis indicated that phosphoproteins associated with RNA splicing, cell-cell adhesion, and mRNA processing were significantly over-represented (p < 10^−5^). Consistent with network analysis performed with global proteomics (Supplemental Figure 4F), a clear reduced phosphorylating of multiple components of the Wnt signaling pathway in *Gli1-Smo^–/–^* mice was observed (Supplemental Figure 6A). Pairwise comparisons between *Gli1-Smo^+/+^* and *Gli1-Smo^–/–^* mice at 1 day after IRI highlighted substantially changed components in the Wnt signaling pathway, such as pGSK3β, pSlc9a3r1, pCtnnd1, pTGFβ1i1, pCcny, and pLeo1 (Figure 4C and Supplemental Figure 6, B and C). In particular, we identified 2 downregulated tyrosine phosphorylation sites (Tyr216 and Tyr279) on GSK-3β in *Gli1-Smo^–/–^* mice. Dephosphorylation on Tyr216 and Tyr279 represses the activity of GSK-3β. Therefore, in *Gli1-Smo^–/–^* mice, inactivated GSK-3β loses its capacity to destabilize β-catenin, a principal protein in the Wnt signaling pathway. This ultimately causes the activation of the canonical Wnt/β-catenin signaling pathway. In addition, the network analysis showed that GSK-3β interacts with other proteins in *Gli1-Smo^–/–^* kidneys as well (Supplemental Figure 6B).

Previously, we and others reported that activation of the Wnt signaling pathway protected against AKI (10, 12, 14, 44). Based on the information provided by the phosphoproteome analysis, we assessed the status of the canonical Wnt signaling pathway in *Gli1-Smo^+/+^* and *Gli1-Smo^–/–^* mice after AKI. qPCR revealed that 9 out of 19 Wnt family members (*Wnt 2, 4, 5A, 5B, 7A, 7B, 9B, 10A,* and *11*) were markedly induced in *Gli1-Smo^–/–^* mice (Figure 4D). In the same kidneys, several Frizzled receptors in the Wnt signaling pathway were also upregulated in *Gli1-Smo^–/–^* mice (Supplemental Figure 7A). Western blots demonstrated that β-catenin, Wnt 1, or Wnt 5A/B proteins were markedly induced in the kidneys of *Gli1-Smo^–/–^* as well as *Pdgfr*-β*-Smo^–/–^* mice after IRI (Figure 4, E and F; quantitative data are presented in Supplemental Figure 7, B and C). IHC staining further confirmed increased expression of β-catenin, Wnt1, Wnt4, or Wnt5A/B in *Gli1-Smo^–/–^* and *Pdgfr*-β*-Smo^–/–^* mouse kidney tubular cells, compared with their corresponding littermate controls (Figure 4G). Of particular interest, we saw impressive inductions of Wnt1, Wnt4, Wnt5A/B in the biopsy specimens from patients with AKI (Figure 4H). Taken together, our data suggest that ablation of Smo in fibroblasts is linked to Wnt signaling pathway activation after ischemic AKI.

### Integrin β1 may link NID1 and the Wnt signaling pathway after AKI.

**Figure 5:**
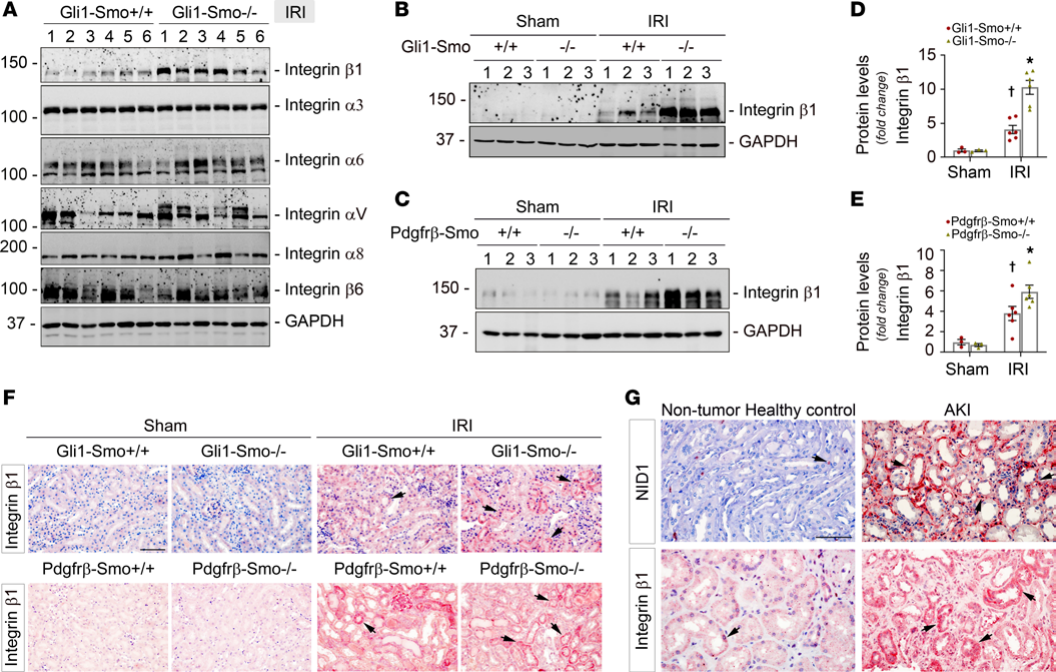
Deleting Smo in fibroblasts affects integrins linked to tubular cells after AKI. (**A**) Western blot assay showing the expression of integrin receptors linked to tubular cells, including integrin β1, α3, α6, αV, α8, and β6, in *Gli1-Smo^+/+^* and *Gli1-Smo^–/–^* kidneys after ischemic AKI at 1 day. Numbers indicate individual animals in a given group. (**B** and **C**) Representative Western blot showing the expression of integrin β1 in *Gli1-Smo^+/+^* and *Gli1-Smo^–/–^* (**B**) or in *Pdgfr*-β*-Smo^+/+^* and *Pdgfr*-β*-Smo^–/–^* (**C**) kidneys at 1 day after ischemic AKI. Numbers indicate individual animals in a given group. Quantitative data are accordingly presented in **D** and **E**. Dots indicate individual animals in a given group (Sham, *n* = 3; IRI, *n* = 6). †*P* < 0.05 versus sham control, *P < 0.05 versus *Gli1-Smo^+/+^* or *Pdgfr*-β*-Smo^+/+^* IRI mice. (**F**) IHC staining showing integrin β1 in *Gli1-Smo^+/+^* and *Gli1-Smo^–/–^* or in *Pdgfr*-β*-Smo^+/+^* and *Pdgfr*-β*-Smo^–/–^* kidneys at 1 day after AKI. Scale bar: 25 μm. (**G**) IHC staining showing NID1 and integrin β1 expression in kidney biopsy specimens from patients with AKI. Arrows indicate positive staining. Scale bar: 25 μm. Graphs are presented as means ± SEM. Differences among groups were analyzed using 1-way ANOVA, followed by the Student-Newman-Keuls test.

Given that specifically deleting Smo in fibroblasts induced interstitial matrix protein NID1 and simultaneously activated the Wnt signaling pathway in tubules after AKI, we sought to explore the possible connection between NID1 and Wnts. As a prominent matrix glycoprotein with potential roles in tissue regeneration (45), NID1 often plays a role in cell interactions with the ECM by activating specific receptors, such as integrins, while integrin receptors could synergize with the Wnt signaling pathway to form a loop to control tissue injury/repair and tumor metastasis (46, 47). So, we systemically evaluated the expression of 6 integrin receptors linked to tubular cells (48, 49), including integrin α3, α6, αV, α8, β1, and β6, in *Gli1-Smo^+/+^* and *Gli1-Smo^–/–^* mice after ischemic AKI. Only integrin β1 was induced in the diseased kidneys between *Gli1-Smo^+/+^* and *Gli1-Smo^–/–^* mice (Figure 5A). Of note, at baseline, integrin β1 was barely detectable in the sham controls. After IRI, compared with *Gli1-Smo^+/+^* or *Pdgfr*-β*-Smo^+/+^* mice, integrin β1 was markedly induced in the kidneys of *Gli1-Smo^–/–^* or *Pdgfr*-β*-Smo^–/–^* mice (Figure 5, B–E). IHC staining consistently showed upregulated integrin β1 in *Gli1-Smo^–/–^* and *Pdgfr*-β*-Smo^–/–^* mouse kidney tubular cells compared with their corresponding littermate controls (Figure 5F). Meanwhile, both NID1 and integrin β1 were increased in the biopsy specimens from patients with AKI (Figure 5G).

**Figure 6:**
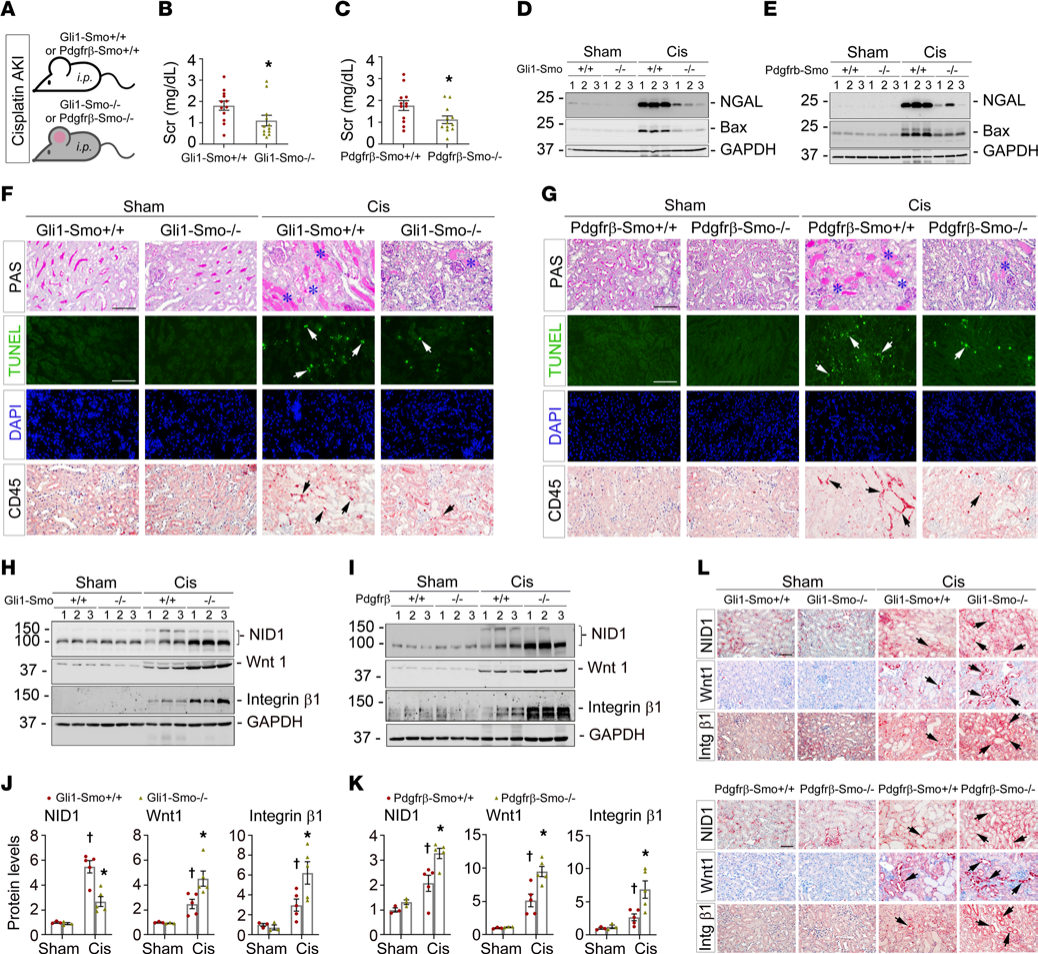
Specific deletion of Smo in fibroblasts mitigates AKI induced by cisplatin. (**A**) Schematic diagram. i.p., intraperitoneal. At 3 days after cisplatin (Cis) injection, (**B**) Serum creatinine (Scr) levels in *Gli1-Smo^+/+^* and *Gli1-Smo^–/–^* mice (*n* = 12). (**C**) Scr levels in *Pdgfr*-β*-Smo^+/+^* and *Pdgfr*-β*-Smo^–/–^* mice (*n* = 12). (**D** and **E**) Western blots assay showing NGAL and Bax protein levels in *Gli1-Smo^+/+^* and *Gli1-Smo^–/–^* (**D**) or in *Pdgfr*-β*-Smo^+/+^* and *Pdgfr*-β*-Smo^–/–^* (**E**) kidneys. (**F**) Periodic Acid–Schiff (PAS) staining showing kidney morphological changes in *Gli1-Smo^+/+^* and *Gli1-Smo^–/–^* mice. Blue asterisks indicate injured tubules. Representative micrographs of TUNEL assay or IHC staining against CD45 in *Gli1-Smo^+/+^* and *Gli1-Smo^–/–^* kidneys. Scale bar: 25 μm. White arrows indicate apoptotic cells adn black arrows indicate CD45^+^ cells. (**G**) Representative micrographs of PAS staining, TUNEL assay, and IHC staining against CD45 in *Pdgfr*-β*-Smo^+/+^* and *Pdgfr*-β*-Smo^–/–^* kidneys. Scale bar: 25 μm. Blue asterisks indicate injured tubules. White and black arrows, respectively, indicate apoptotic cells and CD45^+^ cells. (**H** and **I**) Western blots assay of NID1, Wnt1, and integrin β1 proteins in *Gli1-Smo^+/+^* and *Gli1-Smo^–/–^* (**H**) or in *Pdgfr*-β*-Smo^+/+^* and *Pdgfr*-β*-Smo^–/–^* (**I**) kidneys. Quantitative data are accordingly presented in **J** and **K** (Sham, *n* = 3; Cis, *n* = 5). †*P* < 0.05 versus sham control, *P < 0.05 versus *Gli1-Smo^+/+^* or *Pdgfr*-β*-Smo^+/+^* mice. Dots indicate individual animals in a given group. (**L**) IHC staining showing NID1, Wnt1, and integrin β1 (Intg β1) in *Gli1-Smo^+/+^* and *Gli1-Smo^–/–^* or in *Pdgfr*-β*-Smo^+/+^* and *Pdgfr*-β*-Smo^–/–^* kidneys. Scale bar: 25 μm. Arrows indicate positive cells. DAPI is a nuclear counterstain. For all Western blot panels, numbers indicate individual animals in a given group. Graphs are presented as means ± SEM. Differences among groups were analyzed using 1-way ANOVA, followed by the Student-Newman-Keuls test.

To validate our findings in ischemic AKI, we constructed a separate AKI mouse model induced by cisplatin (Figure 6A). At 3 days after cisplatin injection, compared with the corresponding controls, serum creatinine levels were reduced by 38.7% or 36.8% in *Gli1-Smo^–/–^* or *Pdgfr*-β*-Smo^–/–^* mice, respectively (Figure 6, B and C). Western blot analysis, TUNEL assay, and histological/immunostaining demonstrated improved kidney morphological changes and reduced cell apoptosis and inflammatory cell infiltration in *Gli1-Smo^–/–^* or *Pdgfr*-β*-Smo^–/–^* mice (Figure 6, D–G; quantitative data in Supplemental Figure 8, A–D). Consistent with IRI model, NID1, Wnt1, and integrin β1 were also increased in *Gli1-Smo^–/–^* or *Pdgfr*-β*-Smo^–/–^* mouse kidneys after cisplatin injection, compared with *Gli1-Smo^+/+^* or *Pdgfr*-β*-Smo^+/+^* mice (Figure 6, H–L). These results indicated that tubular-derived integrin β1 is a potential mechanistic clue in elucidating the connection between NID1 and Wnts after various insults caused-AKI.

### Genetic knockdown or pharmaceutical inhibition of Smo in fibroblasts promotes tubular cell survival through NID1 in vitro and ex vivo.

**Figure 7:**
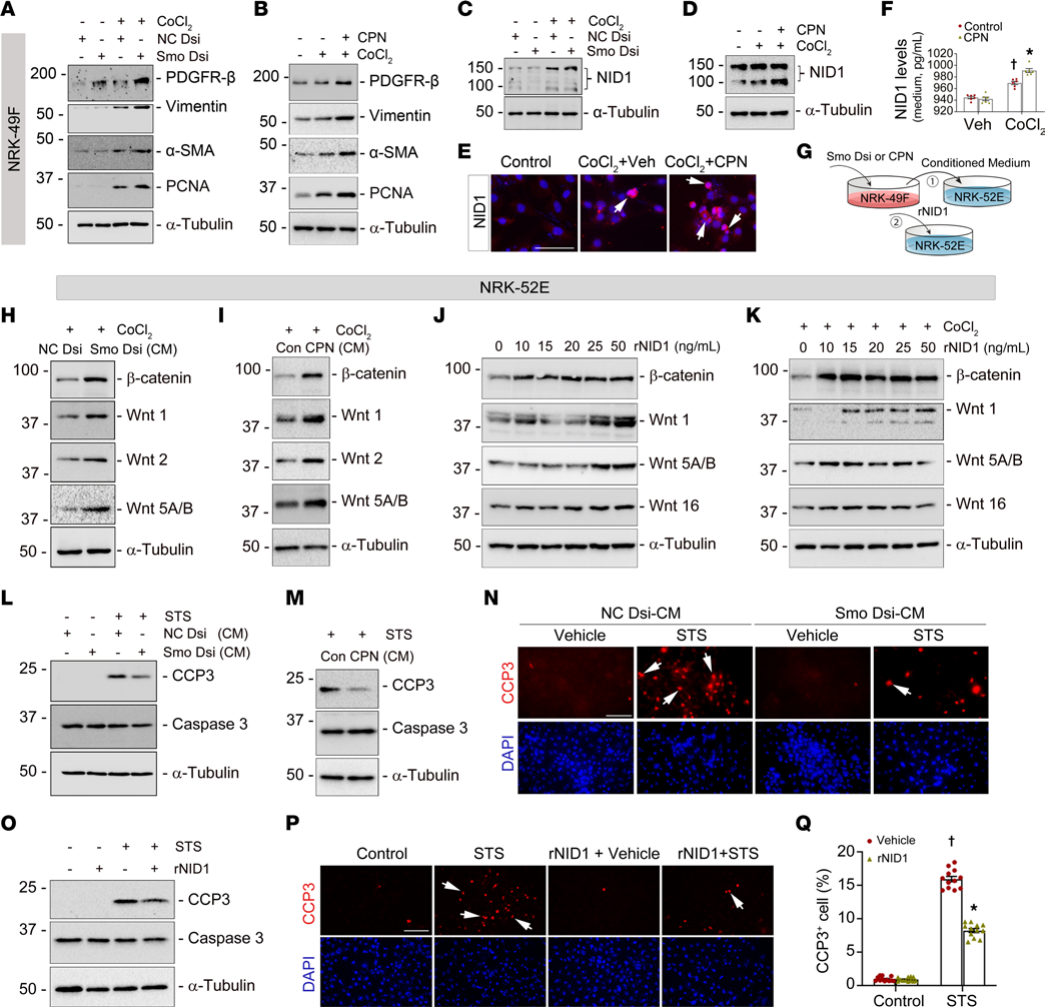
NID1 promotes Wnt components expression and reduces tubular cell death in vitro. (**A**–**D**) Normal rat kidney fibroblasts (NRK-49F) were transfected with Dicer-substrate Smo siRNA (Smo Dsi) or incubated with Smo inhibitor cyclopamine (CPN, 2.5 μM), then subjected to hypoxic stress (CoCl_2_, 400 μM) for 24 hours. Compared to control siRNA (NC Dsi) or vehicles, western blots demonstrating that knockdown (**A**) or inhibition (**B**) of Smo induced Pdgfr-β, vimentin, αSMA, and PCNA expression, and increased NID1 (**C** and **D**). (**E**) Immunofluorescence staining showing Smo inhibition induced NID1 in fibroblasts under hypoxic stress. Scale bar: 25 μm. Arrows indicate positive staining. (**F**) Enzyme-linked immunosorbent assay revealed NID1 concentration after incubation with CPN under hypoxic stress (*n* = 6). (**G**) Schematic diagram. (**H** and **I**) Western blots demonstrating β-catenin, Wnt1, Wnt2, and Wnt5A/B levels after knockdown (**H**) or inhibition (**I**) of Smo under hypoxic stress. (**J** and **K**) Western blots showing that NID1 recombinant protein (rNID1) elevated β-catenin, Wnt1, Wnt5A/B, and Wnt16 in cultured normal rat kidney proximal tubular cells at different dosages under basal conditions (**J**) and hypoxic stress (**K**). (**L**, **M**, and **O**) After stimulation with staurosporine (1 μM) for 3 h, western blots assay showing reduced cleaved-caspase 3 (CCP3) in tubular cells incubated with NID1-enriched CM collected from Smo-knockdown (**L**) or CPN-treated (**M**) fibroblasts or directly treated with rNID1 (**O**). (**N** and **P**) Immunofluorescence staining showed fewer CCP3+ cells after treated with NID1-enriched CM (**N**) or NID1 recombinant protein (**P**). Quantitative data are presented in **Q** (*n* = 3, 4 random images were selected per slide, each dot represents the score of the according image). †*P* < 0.05 versus control, *P < 0.05 versus vehicle after STS. Scale bar: 25 μm. Cells were costained with DAPI. Arrows indicate positive staining. Graphs are presented as means ± SEM. Differences among groups were analyzed using 1-way ANOVA, followed by the Student-Newman-Keuls test.

To further decipher the role of fibroblast-selective Smo in regulating tubular cell survival, normal rat kidney fibroblasts (NRK-49F) were transfected with Dicer-substrate Smo siRNA (Supplemental Figure 9A) or treated with cyclopamine (CPN), a small-molecule inhibitor of Smo. Under hypoxic stress induced by CoCl_2_, knocking down or blocking Smo activity promoted the expression of Pdgfr-β, vimentin, α-SMA, and PCNA proteins in the cultured fibroblasts (Figure 7, A and B). Meanwhile, NID1 protein was induced in the same set of cell lysates (Figure 7, C and D), and immunofluorescence staining of whole cells showed similar results (Figure 7E and Supplemental Figure 9B). Quantitatively, enzyme-linked immunosorbent assay revealed that NID1 concentration was higher in the conditioned medium (CM) collected from the cultured fibroblasts after incubation with CPN under hypoxic stresses compared to vehicles (Figure 7F).

To validate our in vivo findings that loss of fibroblast-selective Smo induced tubular Wnts and promoted cell survival, we performed several in vitro and ex vivo experiments. In vitro, we first treated normal rat kidney epithelial cells (NRK-52E) with NID1-enriched CM collected from Smo-knockdown or CPN-treated fibroblasts (Figure 7G). Under basal conditions, NID1-enriched CM did not cause dramatic changes in the expression of β-catenin, Wnt1, Wnt2, and Wnt5A/B in NRK-52E cells (Supplemental Figure 9, C and D). However, these proteins were markedly induced under hypoxic stress after incubated with NID1-enriched CM (Figure 7, H and I). Second, we applied NID1 recombinant protein to treat NRK-52E at different dosages, as depicted in Figure 7G. NID1 activated β-catenin, Wnt1, Wnt5A/B, and Wnt16 in both basal and hypoxic conditions (Figure 7, J and K). Impressively, either NID1-enriched CM or NID1 recombinant protein possessed the capacity to reduce tubular cell apoptosis. Specifically, less caspase 3 cleavage was detected in NRK-52E cells after treatment with NID1-enriched CM or NID1 recombinant protein after being stimulated with a classic apoptosis inducer, staurosporine (Figure 7, L, M, and O). Immunofluorescence staining for cleaved caspase-3 confirmed these findings (Figure 7, N and P and Supplemental Figure 9F; quantitative data in Figure 7Q and Supplemental Figure 9, E and G).

**Figure 8:**
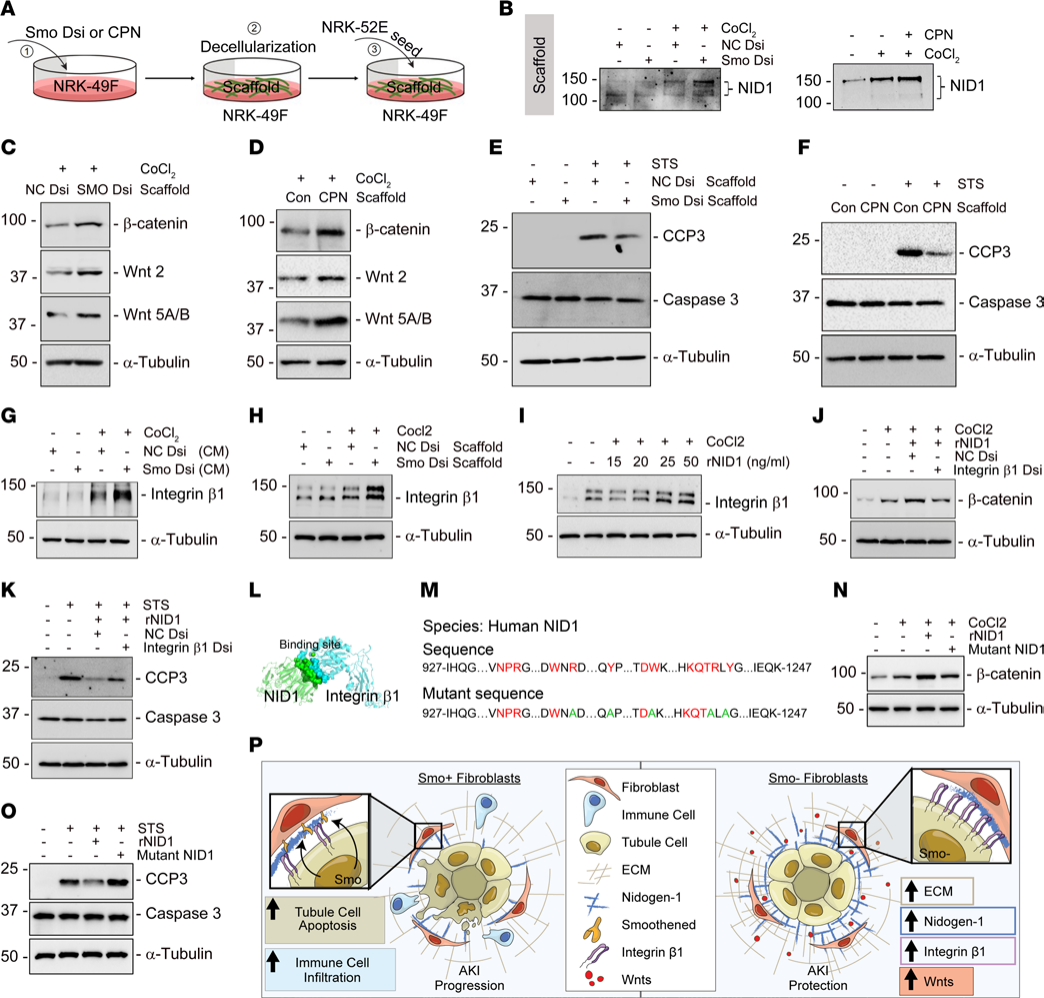
NID1-enriched decellularized fibroblast matrix scaffold activates Wnt components and reduces tubular cell death ex vivo. (**A**) Experimental design. In step 1, normal rat kidney fibroblasts (NRK-49F) were transfected with Dicer-substrate Smo siRNA (Smo Dsi) or cultured with Smo inhibitor CPN to enrich for NID1. In step 2, scaffolds were isolated, and, in step 3, normal rat kidney proximal tubular cells (NRK-52E) were seeded on top of scaffolds. (**B**) Western blots assay showing NID1 protein in decellularized fibroblast matrix scaffold. (**C** and **D**) Western blots assay showing β-catenin, Wnt2, and Wnt5A/B were activated in NRK-52E cells seeded on NID1-enriched matrix scaffold isolated from Smo-knockdown (**C**) or CPN-treated (**D**) fibroblasts under hypoxic stress. (**E** and **F**) Western blots assay showing NID1-enriched matrix scaffold isolated from Smo-knockdown (**E**) or CPN-treated (**F**) fibroblasts reduced cleaved caspase-3 (CCP3) in the seeded NRK-52E cells. (**G**–**I**) Western blot assay showing conditioned medium collected from Smo-knockdown fibroblasts (**G**) or decellularized fibroblast matrix scaffold isolated from Smo-knockdown fibroblasts (**H**) or NID1 recombinant protein (rNID1) increased integrin β1 in NRK-52E cells under hypoxic stress. (**J** and **K**) Under hypoxic stress, knockdown of integrin β1 using Dicer-substrate siRNA (integrin β1 Dsi) repressed β-catenin accumulation after incubation with rNID1 in NRK-52E cells (**J**) and induced CCP3 (**K**). (**L**) Molecular docking analysis showing the binding sites between NID1 and Integrin β1. (**M**) The strategy of designing a mutant form of NID1. (**N** and **O**) Compared with the active form of NID1 human recombinant protein (25 ng/mL), Western blot assay demonstrating that mutant NID1 (25 ng/mL) failed to induce β-catenin expression in NRK-52E cells under hypoxic stress (**N**) and increased CCP3 after staurosporine (1 μ**M**) stimulation (**O**). (**P**) Our model illustrates that loss of fibroblast-selective Smo promotes NID1 to interact with tubular integrin β1 and subsequently activated the Wnt signaling pathway in tubules, forming a favorable microenvironment to protect against AKI.

To better mimic the in vivo microenvironment, we decellularized cultured fibroblasts (Control, Smo-knockdown, or CPN-treated) after hypoxic stress for 24 hours to obtain matrix scaffolds. We then seeded NRK-52E cells onto the scaffold-coated plate (Figure 8A), as we have done in previous studies (50). NID1 was markedly enriched in the decellularized matrix scaffold isolated from Smo-knockdown or CPN-treated fibroblasts (Figure 8B). Under basal conditions, there were minor differences in β-catenin, Wnt 2, and Wnt 5A/B expression in the seeded tubular cells between control scaffolds and NID1-enriched scaffolds (Supplemental Figure 10, A and B). Under hypoxic stress, the NID1-enriched scaffold exhibited an enhanced capacity to activate β-catenin, Wnt 2, and Wnt 5A/B and repress caspase-3 cleavage in NRK-52E cells (Figure 8, C–F). Mechanistically, NID1-enriched CM/matrix scaffold or NID1 recombinant protein activated integrin β1 in the tubular cells (Figure 8, G–I), whereas knockdown of integrin β1 repressed β-catenin accumulation, which resulted in inactivation of the canonical Wnt signaling pathway and, consequently, increased cleaved-caspase 3 levels in tubular cells (Figure 8, J and K and Supplemental Figure 10C). To confirm the above findings, we performed a molecular docking study. A strong binding site with extremely low dock energy between NID1 and integrin β1 was identified; the site formed a dense hydrogen bonding network system to stabilize the bindings (Figure 8L and Supplemental Figure 11, A and B). We then mutated 5 amino acids in NID1 protein sequences that mainly contributed to the binding and substituted them with alanine (Figure 8M and Supplemental Figure 11C). Detailed information is presented in the Supplemental Methods. We then treated NRK-52E cells with mutant or active NID1, and we observed that mutant NID1 failed to induce β-catenin compared with the active NID1 under hypoxic stresses, because it could not bind integrin β1 receptor (Figure 8N). Similar to the result using integrin β1 knockdown approach, mutant NID1 increased cleaved-caspase 3 levels in tubular cells after staurosporine stimulation (Figure 8O). Taken together, our results suggest that inhibiting Smo in fibroblasts liberated NID1 to activate the Wnt signaling pathway in tubules through integrin β1. This, in turn, created a favorable kidney local microenvironment to prevent or mitigate AKI (Figure 8P).

## Discussion

Although the kidney is a vulnerable organ that has limited regenerative capacity, there is consensus that the tubular epithelium can repair damage to the kidney after AKI (2, 51). The mechanisms involved are rigorously debated. For a long time, proximal tubule progenitors were thought to exist in various locations such as bone marrow, the renal interstitium, and the renal papilla (2). In recent years, the concept of kidney perivascular mesenchymal cells contributing to tubule repair in AKI has been increasingly appreciated (3, 4, 7, 11), although many unknowns remain. In this study, combining conditional-KO mice, in vitro and ex vivo models, and systems biology approaches, we illustrated that fibroblasts play an important role in protecting against AKI. Our results indicate that ablation of *Smo* in *Gli1*^+^ or *Pdgfr*-β^+^ fibroblasts (a) mitigates AKI caused by IRI or cisplatin, (b) promotes perivascular mesenchymal cells activation, (c) upregulates NID1 and activates the Wnt signaling pathway in tubules, and (d) reveals that integrin β1 may bridge NID1 and the Wnt signaling pathway in tubules, which may explain reduced tubular cell death and increased cell survival after AKI.

Smo is one of the multispan transmembrane proteins in the core reception system for the Hh pathway (52, 53). It has been well documented that Hh controls cell growth and differentiation through a highly conserved signal transduction pathway that reciprocally travels between producing and responsive cells (20, 27, 54). In the kidney, deleting Shh in producing tubular cells aggravates AKI (4), whereas the current study surprisingly shows that loss of Smo in Hh-responding fibroblasts mitigates tubular cell apoptosis and inflammatory cell infiltration after AKI (Figures 1 and 6). These opposing observations reflect the challenge of elucidating AKI if focused on a single signaling pathway or cell type. What we observed was of particular interest: after AKI, loss of Smo in fibroblasts activated substantial perivascular mesenchymal cells such as non-*Gli1*^+^/*Pdgfr*-β^+^ fibroblasts, pericytes, and mesenchymal stromal cells (Figure 2). The number of proliferative interstitial cells in Smo conditional-KO mice is apparently more than their corresponding littermate controls, indicating interstitial cells’ potential in mitigating AKI. But why did the loss of Smo in fibroblasts in turn activate perivascular mesenchymal cells after AKI? There are 2 possibilities. The first is that, because our conditional-KO mouse models were generated by an inducible Cre-LoxP system in which *Gli1*^+^ or *Pdgfr*-β^+^ fibroblasts are a small fraction of the entire population of perivascular mesenchymal cells (11, 32, 55). Therefore, after AKI, these non-*Gli1*^+^ or non-*Pdgfr*-β^+^ fibroblast subpopulations are instinctively mobilized to rescue themselves and their neighboring cells, such as tubular cells. The second is that these early and overactivated fibroblasts can immediately synthesize matrix proteins and initiate a favorable kidney local microenvironment (KLM) through multiple mechanisms such as cell mechanical changes (56), and by simultaneously recruiting growth factors from other cell types such as tubule-derived Wnts and Hh (Figures 3 and 4). These growth factors may further fibroblast activities by a transient feedback loop. However, defining the subpopulation of fibroblast is a challenge due to the highly heterogeneous nature of cells. The current study still lacks analysis at the single-cell level to fully fill the gap in elucidating the phenomena of fibroblast overactivation in *Gli1-Smo^–/–^* and *Pdgfr*-β*-Smo^–/–^* mouse kidneys after AKI.

AKI is a refractory clinical syndrome. Therefore, its repair process is certainly to involve multicellular and/or noncellular components. Consistent with the enhanced activation of perivascular mesenchymal cells in *Gli1-Smo^–/–^* or *Pdgfr*-β*-Smo^–/–^* kidneys (Figure 2), global proteomics confirmed that ECM is a key cellular compartment where differentially expressed proteins are distributed after ischemic AKI (Figure 3). As a 3-dimensional noncellular component in the kidney, ECM provides the biophysical scaffolding for the kidney and biochemical or mechanical microenvironment to control cell homeostasis and differentiation (57–59). The number of markedly upregulated ECM proteins in *Gli1-Smo^–/–^* mice is nearly 3.5-fold more than in their littermate controls (Figure 3). These quantitative analyses suggest perivascular mesenchymal cells synthesized an appropriate amount of ECM to reconstruct kidneys under acute ischemic stress. Among these identified ECM markers, NID1 is one of the most prominent matrix proteins increased in *Gli1-Smo^–/–^* and *Pdgfr*-β*-Smo^–/–^* mice (Figure 3). NID1, formerly known as entactin, is an essential structural component of the basement membrane and ECM. The basement membrane is a specialized layer of ECM components in the kidney that plays a central role in maintaining organ function (60). Our data showed that NID1 is predominantly localized in kidney interstitium after AKI (Figure 3). Thus far, most studies have focused on secretion of NID1 preventing cancer metastasis. Sporadic studies have reported that NID1 mitigates ischemia and promotes heart survival and regeneration after injury (45, 61). Consistent with the positive roles NID1 plays in treating various diseases, our data provides additional evidence for a role of NID1 in AKI and kidney repair as well.

Another interesting finding of our study is that Smo physically binds NID1 to form a protein-protein complex. As a frizzled-class G-protein-coupled receptor (GPCR), Smo contains 2 distinct ligand-binding sites: one in its heptahelical transmembrane domain (TMD) and another in the extracellular cysteine-rich domain (62). The TMD binding site is known to bind the small-molecule Smo-specific inhibitor CPN (63). In comparison, whether a GPCR could physically link to macromolecules like NID1, integrate into the basement membrane, and subsequently give them unique functions in diseased kidneys is unclear. We showed through immunoprecipitation that Smo binds to NID1 (Figure 3). Therefore, in the absence of Smo, NID1 protein would be released (Figure 7) and deposited in the neighboring epithelial basement membranes. Basement membranes are essential ECM structures shaped by the networks of collagen type IV and laminins, both of which are linked by NID1 (45). This strategic subcellular localization of NID1 enables it to mediate cell attachment and communications between cells and ECM (64–66). We also demonstrated in our in vitro and ex vivo systems that increased NID1 reduced tubular cell apoptosis and promoted cell survival (Figure 7 and 8).

Considering the visual pathological effects of AKI repair, it is undeniable that the fibroblast-ECM-tubule interaction is a standard process of building a favorable KLM, which is complex, heterotypic, and dynamic (67). We need to ask whether a NID1-enriched KLM plays a role in repairing AKI. In this study, we utilized a decellularized scaffold that retains fibroblasts’ overall architecture to closely imitate the in vivo matrix microenvironment. The NID1-enriched scaffold activated the Wnt signaling pathway and reduced tubular cell death (Figure 8). Most importantly, these biological capacities of NID1 in protecting against AKI were accomplished by strongly binding to the tubular integrin β1 receptor, thereby forming a loop with Wnts signals in tubules (Figures 5, 6, and 8). Our global and phosphoproteomics data coincidentally provided us with solid information that the Wnt signaling pathway was dramatically activated in *Gli1-Smo^–/–^* mice after AKI. As we know, at the early phase of AKI, most Wnt family proteins are activated in tubules and exhibit a protective role in preserving kidney function (12). Of note, canonical Wnt signaling is controlled by protein phosphorylation and dephosphorylation. Here, we identified the downregulated tyrosine phosphorylation sites (Tyr216 and Tyr279) on GSK-3β, destabilizing β-catenin in *Gli1-Smo^–/–^* mice after AKI (Figure 4). Phosphorylation-dependent β-catenin degradation is a key step in turning off the Wnt signaling pathway (68, 69). Besides GSK-3β, the extent of phosphorylation of dishevelled protein and low-density lipoprotein receptor–related proteins, the activities of the Axin–scaffold phospho-destruction complex, and the modulation of kinases such as nemo-like kinase and homeodomain-interacting protein serine/threonine kinases can affect gene-specific β-catenin recruitment and its transcription (70). In addition, our phosphoproteomics identified cell-cell adhesion as a markedly changed biological event in *Gli1-Smo^–/–^* mice after AKI. This cell-cell adhesion is worth further investigation, given that the connection between NID1 and integrin β1 demonstrated in the current system.

Our study has some limitations. First, the core reception system for Hh signaling includes 2 multispan transmembrane proteins—Ptc and Smo—and Hh reciprocally controls them. Ptc inhibits Smo in the absence of Hh. In the presence of Hh, Hh binds Ptc to release its inhibition of Smo, leading to Smo phosphorylation and activation. However, whether loss of fibroblast-selective Smo influences Ptc in AKI repair was not studied here. Second, we observed increased fatty acid oxidation in *Gli1-Smo^–/–^* mice after AKI, but how disrupting Smo affects energy metabolism after AKI remains unclear. Third, the *Gli1* or *Pdgfr*-β promoter in the Cre-LoxP system limited the population of the target fibroblasts. So, we cautiously described the *Gli1^+^* or *Pdgfr*-β*^+^* fibroblasts in relatively broad terms by encompassing perivascular mesenchymal cells, pericytes, and fibroblasts. More robust genetic animal models and omics analyses at the single-cell level are needed to define these cell populations in AKI. Lastly, while this study posits that *Gli1^+^* or *Pdgfr*-β*^+^* fibroblast-Smo deletion results in activation of non-*Smo^–/–^* fibroblasts, upregulation of NID1, and activation of the Wnt signaling pathway in tubule epithelial cells, we did not mechanistically test how non-*Smo^–/–^* fibroblasts were activated. Still, our results provide some mechanistic insight into the link between Smo, NID1, Wnt signaling, and AKI mitigation, and offer additional pathways that we will pursue in future experiments.

In summary, using a multidisciplinary approach, we report that loss of Smo in *Gli1^+^* or *Pdgfr*-β*^+^* fibroblasts protects against AKI through ECM-cell interactions involving multiple signaling pathways. We highlight the importance of creating a favorable tissue microenvironment in determining AKI prognosis. Our findings provide preclinical stage strategies to understand AKI better and shed light on effectively restoring kidney functions.

## Methods

The detailed information of methodology, nucleotide sequences of the primers (Supplemental Table 4), chemical, biological reagents, and antibodies (Supplemental Table 5) is presented in the Supplemental Methods.

### Sex as a biological variable.

Our AKI study exclusively examined male mice because female mice represented greater resistance than male mice against renal IRI and cisplatin-induced toxicity. Human kidney biopsy samples were obtained from both men and women. It is unknown whether the findings in male mice are relevant to female mice.

### Statistics.

All data were expressed as mean ± SEM if not specified otherwise in the legends. Statistical analysis of the data was performed using GraphPad Prism 9 (GraphPad Software, San Diego, California, USA). Comparison between 2 groups was made using a 2-tailed Student’s *t* test or the Rank Sum test if data failed a normality test. Statistical significance for multiple groups was assessed by 1-way ANOVA, followed by the Student-Newman-Keuls test. Results are presented in dot plots, with dots denoting individual values. *P* < 0.05 was considered statistically significant.

### Study approval.

All animal experiments were performed in accordance with institutional and federal guidelines and approved by the Institutional Animal Care Committee of the University of Pittsburgh, School of Medicine (Protocol number: 16048123) and the University of Connecticut, School of Medicine (Protocol number: AP-200105-0923).

### Data availability.

Data are available in the Supporting Data Values file. Raw Mass Spectrometry data were deposited in MassIVE with the data set identifier MSV000088609. (doi:10.25345/C5NS23).

## Authors contributions

YG and DZ conceived the project. YG and DZ wrote and revised the manuscript. YG, HF, ZP, JT, YQ, and YW performed most of the in vivo, ex vivo, in vitro studies involving mRNA expression analysis, Western blotting assay, immunostaining, and imaging. Yansheng Liu, YY, and Yi-Han Lin performed proteomic analysis. WM and PY performed the docking study. YG, CB, GH, DLK, YW, Youhua Liu, and DZ edited the manuscript. DZ supervised the entire project.

## Supplementary Material

Supplemental data

Unedited blot and gel images

Supplemental table 1

Supplemental table 2

Supplemental table 3

Supporting data values

## Version 1 05/07/2024

In-Press Preview

## Version 2 07/01/2024

Electronic publication
