# Supplementary material for: Fibroblast expression of transmembrane protein smoothened governs microenvironment characteristics after acute kidney injury
Source: J Clin Invest. 2024 May 7;134(13):e165836. doi: 10.1172/JCI165836 (PMC11213467; doi:10.1172/JCI165836)
Supplement: Supplemental data [file jci-134-165836-s093.pdf]

Fibroblast expression of transmembrane protein smoothened governs microenvironment characteristics after acute kidney injury

Yuan Gui, Haiyan Fu, Zachary Palanza, Jianling Tao, Yi-Han Lin, Wenjian Min, Yi Qiao, Christopher Bonin, Geneva Hargis, Yuanyuan Wang, Peng Yang, Donald L. Kreutzer, Yanlin Wang, Yansheng Liu, Yanbao Yu, Youhua Liu, Dong Zhou

Supplementary Methods

Sex as a biological variable

Our AKI study exclusively examined male mice because female mice represented greater resistance than male mice against renal ischemia-reperfusion injury and cisplatin-induced toxicity. Human kidney biopsy samples were obtained from both male and female subjects. It is unknown whether the findings in male mice are relevant to female mice.

Mice and genotyping

Heterozygous *Smo*-floxed mice ($\text{Smo}^{\text{tm2Amc/J}}$) were purchased from the Jackson Laboratory (Stock #004526; Bar Harbor, ME). During the revision stage of this manuscript, *Smo*-floxed mice were generously gifted by Dr. Anna Mae Diehl at Duke University (Durham, NC). Transgenic mice [$\text{Gli1}^{\text{tm3(cre/ERT2)Alj/J}}$] that express Cre recombinase fused to a triple mutant form of the human estrogen receptor under the control of endogenous Gli1 promoter/enhancer elements were obtained from the Jackson Laboratory (Stock #007913; Bar Harbor, ME) as well, as previously described[S1]. Similarly, Pdgfrβ-P2A-CreER^{T2} (B6.Cg-$\text{Pdgfr}\beta^{\text{tm1.1(Cre/ERT2)Csln}}$/J) mice express Cre-$\text{ER}^{\text{T2}}$ directed by the murine platelet-derived growth factor receptor β polypeptide (Pdgfrβ) promoter were also purchased from the Jackson Laboratory (Stock #030201; Bar Harbor, ME). By mating *Smo*–floxed mice with Gli1-CreER^{T2} or Pdgfrβ-P2A-CreER^{T2} transgenic mice, conditional knockout mice in which *Smo* gene was specifically disrupted in kidney Gli1+ or Pdgfrβ+ fibroblasts (genotype $\textit{Smo}^{\text{fl/fl}}$, Cre ±) were created. These mice were crossbred with homozygous *Smo*–floxed mice (genotype $\textit{Smo}^{\text{fl/fl}}$) to generate offspring with 50% Gli1-Smo-/- or Pdgfrβ-Smo-/- mice and 50% control mice (Gli1-Smo+/+ or Pdgfrβ-Smo+/+) within the same litters. A routine PCR protocol was used for genotyping of tail DNA samples with the following primer pairs: Cre transgene (Gli1-CreER^{T2}), 5'-GCG-GTC-TGG-CAG-TAA-AAA-CTA-TC-3' and 5'-GTG-AAA-CAG-CAT-TGC-TGT-CAC-TT-3'; Cre transgene

(Pdgfrβ-P2A-CreER^{T2}), 5'-AGC-TTG-TGG-CAG-TGT-AGC-TG-3', 5'-ACA-TGT-CCA-TCA-GGT-TCT-TGC-3', and 5'-CCA-CCT-TGA-ATG-AAG-TCA-ACA-C-3'; and *Smo* genotyping, 5'-CGC-ACC-GGT-CGC-CTA-AGT-AGC-3' and 5'-GGC-GCT-GTG-AGG-CCC-AGG-C-3'. All animals were born normally at the expected Mendelian frequencies. At baseline, they were normal in size and did not display any gross physical or behavioral abnormalities. All animals were housed in the universities' animal facilities. The room temperature is maintained between 69° to 72° F. Light timers are set for 12-hour light/12-hour dark. Animal experiments were approved by the Institutional Animal Care and Use Committee at the University of Pittsburgh School of Medicine and the University of Connecticut School of Medicine.

Mouse models of AKI

As previously described[S1], 8- to 10-week-old Gli1-Smo+/+ and Gli1-Smo-/- or Pdgfrβ-Smo+/+ and Pdgfrβ-Smo-/- mice were intraperitoneally administrated tamoxifen (T5648, Sigma-Aldrich, St. Louis, MO) at 30 mg/kg for 5 consecutive days to induce target *Smo* gene ablation. After 1 week of washout, these mice were subjected to IRI or Cisplatin injection following an established protocol[S1-3]. In brief, after mice were anesthetized, a midline abdominal incision was made, and bilateral renal pedicles were clipped for 30 minutes using microaneurysm clamps. The mouse body temperature was maintained between 36°C and 37.5°C using a temperature-controlled heating system during the ischemic period. Mice were euthanized at 1 day after IRI, and serum and kidney tissues were collected for various analyses. The cisplatin-induced AKI model involved a single intraperitoneal injection of cisplatin at 30 mg/kg body weight, and then mice were sacrificed 3 days after injection.

Determination of Serum Creatinine

Serum was collected from mice at 1 day after IRI. Serum creatinine level was determined using a QuantiChrom creatinine assay kit, according to the protocols specified by the manufacturer (BioAssay Systems, Hayward, CA). The serum creatinine level was expressed as milligrams *per* 100 ml (dL). Of note, the baseline serum creatinine levels of Gli1-Smo+/+, Gli1-Smo-/-, Pdgfrβ-Smo, and Pdgfrβ-Smo-/- mice (n=6/group) were measured by high-performance liquid chromatography at the George O'Brien Kidney Center at Yale University.

Mouse Blood Pressure Measurement

Systolic and diastolic blood pressures at daytime and night were measured respectively in untreated conscious Gli1-Smo+/+, Gli1-Smo-/-, Pdgfrβ-Smo, and Pdgfrβ-Smo-/- mice (n=6/group) using a noninvasive tail-cuff device (Visitech Systems, BP2000 Series II, Apex, NC), as described elsewhere[S4,5]. The blood pressure measurement was conducted in a designated quiet area (22 ± 2°C). Mice were placed in the specialized holder for 10 –15 minutes before the measurement to acclimate to their surroundings. Heating pads, supplied as part of the Visitech Systems BP2000 system, were preheated to 37°C. Before initial baseline measurements, all animals underwent one-week (twice/day: daytime and night) training.

Human Kidney Biopsy Specimens

Human kidney specimens were obtained from diagnostic kidney biopsies performed at the Presbyterian Hospital of the University of Pittsburgh Medical Center. Nontumor kidney tissue from patients with renal cell carcinoma who underwent nephrectomy was used as normal controls. All patients in the presented study signed the informed consent forms before they underwent kidney biopsy or nephrectomy. The Institutional Review Board approved all studies involving human kidney sections at the University of Pittsburgh and the University of Connecticut School of Medicine.

Histology and Immunohistochemical Staining

Paraffin-embedded mouse kidney sections (3 µm thickness) were prepared by a routine procedure. The sections were stained with periodic acid–Schiff staining reagents by standard protocol. Immunohistochemical staining was performed according to the established protocol as described previously[S6]. After incubation with primary antibodies at 4°C overnight, the slides were then stained with HRP-conjugated secondary antibody (Jackson ImmunoResearch Laboratories, West Grove, PA). Non-immune normal IgG was used to replace primary antibodies as a negative control, and no staining was visible. Slides were viewed under an Olympus BX43 microscope equipped with a digital camera (Allentown, PA). The image quantification was independently performed by two experienced technicians. The detailed information of antibodies used is presented in Supplementary Table S5.

Immunofluorescence staining

Kidney cryosections were fixed with 3.7% paraformaldehyde for 15 min at room temperature. NRK-

49F and NRK-52E cells cultured on coverslips were fixed with cold methanol:acetone (1:1) for 10 min at room temperature. After blocking with 10% donkey serum for 1 hour, the slides were immunostained with primary antibodies. These slides were then stained with Cy2- or Cy3-conjugated secondary antibody (Jackson ImmunoResearch Laboratories, West Grove, PA). Slides were viewed under an Olympus BX43 microscope equipped with a digital camera or an Olympus FluoView 1000 confocal microscope. The image quantification was independently performed by two experienced technicians. The detailed information of antibodies used is presented in Supplementary Table S5.

Detection of apoptotic cells

Apoptotic cell death was determined by using TUNEL staining with a DeadEnd Fluorometric Apoptosis Detection System (Promega, Madison, WI) or immunostaining with anti-cleaved caspase 3 (#9664S, Cell Signaling Technology, Danvers, MA), as described previously[S7-9]. The cell number counting for the positive apoptotic cells was independently performed by two experienced technicians.

Western Blot Analysis

Kidney tissues were lysed with radioimmune precipitation assay (RIPA) buffer containing 1% NP-40, 0.1% SDS, 100 μg/ml PMSF, 1% protease inhibitor cocktail, and 1% phosphatase I and II inhibitor cocktail (Cell Signaling Technology, Danvers, MA) in PBS on ice. The supernatants were collected after centrifugation at 13,000×g at 4°C for 15 min. Protein expression was analyzed by western blot analysis as described previously[S10]. The detailed information of antibodies used is presented in Supplementary Table S5.

Quantitative Real-Time Reverse Transcription PCR (qRT-PCR)

Total RNA isolation and qRT-PCR were carried out by procedures described previously[S10]. Briefly, the first strand cDNA synthesis was carried out using a reverse transcription system kit according to the instructions of the manufacturer (Promega). qRT-PCR was performed on an ABI PRISM 7000 sequence detection system (Applied Biosystems, Foster City, CA). The mRNA levels of various genes were calculated after normalizing with β-actin. Primer sequences used for amplifications are presented in Supplementary Table S4.

Global Proteomics and Phosphoproteomics Sample Preparation

Kidney tissues were processed following a shotgun proteomics workflow published previously[S11]. In brief, Gli1-Smo+/+ and Gli1-Smo-/- mice kidneys were lysed using SDS buffer (4% SDS, 50mM EDTA, 20mM DTT, 2% Tween 20, 100mM Tris-HCl, pH 8.0) and sonication (Misonix Sonicator 3000 Ultrasonic Cell Disruptor). Around 0.5 mg proteins were digested using a glass fiber-based Suspension Trapping (STrap) approach[S12]. An aliquot (50 µg) of the digests were directly desalted using spinnable StageTip protocol[S13]. The rest were processed with phosphorylation enrichment using TiO_2 beads as described previously[S11]. Desalted peptides were stored at -80 °C until further analysis.

LC-MS/MS analysis

The LC-MS/MS analysis was performed using an Ultimate 3000 nanoLC and Q Exactive mass spectrometer system (Thermo Scientific). Peptides were separated on in-house packed capillary columns (19 cm length × 75 µm ID; Reprosil C18, 3 µm) at a flow rate of 200 nl/min. The mobile phases were (A) 0.1% formic acid in water and (B) 0.1% formic acid in acetonitrile. Gradient elution was performed as follows: 1 to 35% B in 180 min, 35 to 80% B in 10 min, stay at 80% for 5 min, and then back to 2% B in 5 min followed by 20 min equilibration. Spray voltage was 1700 V and the scan range was m/z 350-1800. The top 10 most intense precursor ions were selected for MS/MS using HCD at 30% energy with an automatic gain control value of 1e5, a maximum injection time of 120 ms, and detection at a mass resolution of 60,000 at m/z 200 in the orbitrap analyzer. MS/MS scans were acquired with an automatic gain control value of 5e4 and a maximum injection time of 120 ms. Dynamic exclusion was set for 30 s with a 10 ppm gate with detection at a mass resolution of 15,000 at m/z 200 in the Orbitrap analyzer.

Protein identification and quantitation.

MaxQuant-Andromeda software suite (version 1.6.3.4) was used for protein identification and quantitation with most of the default parameters[S14]. The mass spectra were searched against a mouse database (17,038 sequences; Reviewed only; version March 2019) downloaded from UniProt Knowledgebase (https://www.uniprot.org/). For global proteome analysis, the following parameters were applied: trypsin as enzyme with two missed cleavage sites; 10 ppm and 20 ppm mass tolerances for precursor and fragments, respectively; protein N-terminal acetylation and methionine oxidation as variable modifications; cysteine carbamidomethylation as a fixed modification; peptide length with at

least 7 amino acids. False discovery rate (FDR) was set at 1% for both proteins and peptides. The MaxQuant output (proteinGroups.txt) was log2-transformed prior to downstream data analyses such as missing value imputation, Hierarchical clustering, Principal Component Analysis (PCA), t-tests, correlation, and volcano plots, which were performed in the Perseus environment using default parameters (version 1.6.2.3). For phosphoproteome analysis, phosphorylation at serine, threonine, and tyrosine was set as an additional variable modification during MaxQuant database search. The cutoff of phosphosite probability estimated by MaxQuant was required to be 0.75 or higher. The MaxQuant output file (Phospho (STY) Sites.txt) was utilized for further bioinformatics analyses and were performed in the Perseus environment as well.

Gene Ontology (GO) and Kyoto Encyclopedia of Genes and Genomes (KEGG) pathway analyses were performed using DAVID Bioinformatics Resources 6.8. For protein network analysis, the StringApp was employed in the Cytoscape environment (version 3.8.0) [S15]. The interaction score was set to 0.9, the highest confidence cutoff, to retrieve potential interactions. The 'Load Enrichment Data' option was enabled during this process to retrieve functional enrichment (minimum significance threshold FDR 0.05) for the STRING network, as we described previously[S11].

Cell culture and treatment

NRK-49F and NRK-52E cells were obtained from the American Type Culture Collection (ATCC, Manassas, VA). Cells were maintained as described previously[S3,9,16]. For conditioned media (CM) collections, under $CoCl_2$-induced hypoxic stress, NRK-49F cells were incubated with CPN (2.5μM) or transfected with Dicer-substrate smo siRNA (rn.Ri.Smo.13) for 24 h and then cultured with serum-free media for additional 24 h. The cultured medium was harvested and centrifuged (3000 rpm for 10 min at 4 °C). The supernatant was aliquoted and stored at -80°C for subsequent experiments. Serum-starved NRK-52E cells were then treated with the CM or Nidogen-1 (NID1) recombinant protein (mutant and active forms) or transfected with Dicer-substrate integrin β1 siRNA (rn.Ri.itgb1.13) under STS (S4400) or $CoCl_2$ (232696; Sigma, St Louis, MO) stress. All in vitro experiments were repeated three times at least.

For primary fibroblasts culture, the cells isolated from the Gli1-Smo+/+, Gli1-Smo-/-, and Pdgfrβ-Smo+/+, Pdgfrβ-Smo-/- mice kidneys (about 10 days old) were cultured with EMEM containing 10% FBS, as we previously described[S9]. *Smo* gene was deleted in the primary fibroblasts

using (Z)-4-Hydroxytamoxifen (H7905, Sigma, St. Louis) for 5 days.

Enzyme-linked immunosorbent assay (ELISA)

The rat NID-1 (MBS2886242) Elisa kit was purchased from MyBioSource, Inc (San Diego, CA). This assay employs the quantitative sandwich enzyme immunoassay technique. Antibody specific for NID1 has been pre-coated onto a 96-well strip plate. We prepared 7 wells for standard, 1 well for blank and added 100 μl each of standard, blank, and sample dilutions into the appropriate wells. The plate was then incubated at 37°C for 2 hours. Liquid from each well was removed and 100μl of Detection Reagent A working solution was added to each well. The plate was incubated again at 37°C for 1 hour. After washing, 100Ml of Detection Reagent B working solution was added to each well and the plate was incubated for 30 minutes at 37°C. Following a wash to remove any unbound reagent, 90 μl of substrate solution was added to the wells, and color developed in proportion to the amount of NID1 bound in the initial step. The color development was stopped, and the intensity of the color was measured immediately using a microplate reader set to 450 nm.

ZDOCK and RDOCK algorithms

The crystal structure of Smo was downloaded from RCSB Protein Data Bank (PDB code 4JKV), and the predicted structure of NID1 is downloaded at AlphaFold Protein Structure Database (https://www.alphafold.ebi.ac.uk/). The Dock Proteins (ZDOCK) and Refine Docked Proteins (RDOCK) protocols of Discovery Studio 2021 (DS2021, Accelrys, CA, USA) were conducted to the virtual protein-protein interactions. The ZDOCK protocol provides rigid body docking of two protein structures using the ZDOCK algorithm[S17]. RDOCK optimizes and scores a set of docked protein poses from ZDOCK via a CHARMm-based procedure[S18]. RDock energy, an energy scoring function, was employed to score these binding protein complexes. For RDock results, the RDock energy (E_RDock) is divided into two parts as the equation. E_sol represents the desolvation energy based on the Atomic Contact Energy. E_elec2 denotes the Electrostatic energy after second minimization with ionic residues in charged state, and β is a scaling factor, set as default value 0.9[S19]. The lower the RDOCK energy of the complex, the more likely it is in the optimal conformation. Equation: E_RDock = E_sol + β*E_elec2

Co-immunoprecipitation

Co-immunoprecipitation was carried out using an established method[S7]. Briefly, NRK-49F cells stimulated with CoCl2 were lysed on ice in 1 ml non-denaturing lysis buffer that contained 1% Triton X-100, 0.01 mol/l Tris-HCl (pH 8.0), 0.14 mol/l NaCl, 0.025% NaN3, 1% protease inhibitors cocktail, and 1% phosphatase inhibitors cocktail I and II (Sigma). After preclearing with normal IgG, cell lysates were incubated overnight at 4°C with 2 mg of anti-Smo (sc-166685) or anti-NID1 (sc-33706; Santa Cruz Biotechnology, Dallas, TX), followed by precipitation with 100 ml of protein A/G Plus-agarose for 3h at 4°C. The precipitated complexes were separated by SDS–polyacrylamide gel electrophoresis and immunoblotted with specific antibodies against NID1 (ab254325, abcam, Waltham, MA) or Smo (sc-166685, Santa Cruz Biotechnology, Dallas, TX), respectively.

Fibroblast decellularized ECM scaffold preparation

Serum-starved NRK-49F cells under $CoCl_2$ induced-hypoxia stress were treated with or without CPN or transfected with Dicer-substrate smo-siRNA for 24 h. Cells were then treated with EGTA (#3889; Sigma, St Louis, MO) (0.5 mM, PH=7.4) in calcium-free PBS, followed by shaking at 4°C for 1h. The treatment was repeated 3-4 times until all cells were lifted from their underlying matrix. The fibroblast decellularized ECM scaffold was rinsed with PBS and then stored at 4°C for further experiments, as previously described[S20]. Some ECM scaffolds were collected by scraping with a rubber policeman in loading buffer for western blot assay to detect NID-1 content. All ex vivo experiments were repeated three times at least.

Molecular docking study

Amino acid sequences for human Nidogen-1 (NID1) and Integrin β1 (ITGB1) were retrieved from the UniProt databank with ID P14543 and P09055, respectively. Currently, the NID1 and ITGB1 protein crystal structures were not resolved in the RCSB PDB protein crystal database. However, the crystal structures with high resolutions could be used as homology modeling templates to construct 3D for NID1 (1gi4/90.91%) and ITGB1 (7nxd/92.23%), respectively.

The following three steps (blind docking and precise docking) were implanted to explore possible binding interface between NID1 and ITGB1. Rosetta software was used for docking simulations[S21]. The docking protocol began with a rigid-body blind docking in which all heavy atoms of protein NID1 and ITGB1 were strictly position restrained. In this step, we randomly placed the proteins roughly

facing each other, and at least 2,000 docking conformations were obtained for further analysis. The rigid docking conformations were clustered by using root-mean-square deviation (RMSD) values, and finally the clustered position with highest ranking ratio was selected as a potential binding spot. Binding spots far away from the others were deleted manually. Using this approach, we were able to achieve an acceptable-quality prediction for the NID1 "binding pocket" of ITGB1. Rosetta software is most accurate when docking locally. In precise docking, the aptamer was randomly placed (within ~10 Å) within the binding pockets, and then the input structure of the aptamer was perturbed by 3 Å translation and 8° rotation before the start of every individual simulation. During precise docking, the side chains of the protein residues at the binding pocket and the complete NID1 were allowed to move. Finally, a maximum number of 100 conformers were considered, and the conformation with the lowest binding energy in two binding spots were selected for final molecular dynamics simulations.

To check the NID1-ITGB1 complex stability, the best docking result in each binding spots were employed for molecular dynamics (MD) simulations. It was carried out by AMBER software (version 16), using AMBER ff99SB force field for complex[S22]. Hydrogen atoms were added to the initial NID1-ITGB1 complex model using the leap module, setting ionizable residues as their default protonation states at a neutral PH value. The complexes were both solvated in a cubic periodic box of explicit TIP3P water model that extended a minimum 10 Å distance from the box surface to any atom of the solute. The particle mesh Ewald (PME) method for simulation of periodic boundaries was used to estimate the long-range electrostatic interactions with a cutoff of 10.0 Å. All bond lengths were constrained using the SHAKE algorithm and integration time step was set to 2fs using the Varlet leapfrog algorithm. To eliminate possible bumps between the solute and the solvent, the entire systems was minimized in two steps. Firstly, the complex was restrained with a harmonic potential of the form k (Δx)2 with a force constant k =100 kcal/mol-1Å-2. The water molecules and counter ions were optimized using the steepest descent method of 2,500 steps, followed by the conjugate gradient method for 2,500 steps. Secondly, the entire system was optimized by using the first step method without any constraint. These two minimization steps were followed by annealing simulation with a weak restraint (k=100kcal/mol-1Å-2) for the complex and the entire system was heated gradually in the NVT ensemble from 0 to 298K over 500ps. After the heating phase, a 10ns MD simulation was performed under 1atm. The constant temperature was selected at 298K with the NPT ensemble. Constant temperature was maintained using the Langevin thermostat with a collision frequency of 2ps-1. The

constant pressure was maintained employing an isotropic position scaling algorithm with a relaxation time of 2ps. RMSD values were tested to monitor the conformation fluctuations of the NID1-ITGB1 complex. Based on the final 10ns MDs trajectory, 3000 snapshots were extracted from the last 3ns trajectory for the final average structure of the complex.

Statistics

All data were expressed as mean ± SEM if not specified otherwise in the legends. Statistical analysis of the data was performed using GraphPad Prism 9 (GraphPad Software, San Diego, CA). Comparison between two groups was made using a two-tailed Student's t-test or the Rank Sum Test if data failed a normality test. Statistical significance for multiple groups was assessed by one-way ANOVA, followed by the Student-Newman-Keuls test. Results are presented in dot plots, with dots denoting individual values. Exact P values are presented for all dot plots. $P < 0.05$ was considered statistically significant.

Supplementary References

S1 Zhou, D. *et al.* Fibroblast-Specific beta-Catenin Signaling Dictates the Outcome of AKI. *J Am Soc Nephrol* **29**, 1257-1271, doi:10.1681/ASN.2017080903 (2018).

S2 Zhou, D. *et al.* Early activation of fibroblasts is required for kidney repair and regeneration after injury. *FASEB J* **33**, 12576-12587, doi:10.1096/fj.201900651RR (2019).

S3 Gui, Y. *et al.* Calponin 2 regulates ketogenesis to mitigate acute kidney injury. *JCI Insight*, doi:10.1172/jci.insight.170521 (2023).

S4 Boe, A. E. *et al.* Plasminogen activator inhibitor-1 antagonist TM5441 attenuates Nomega-nitro-L-arginine methyl ester-induced hypertension and vascular senescence. *Circulation* **128**, 2318-2324, doi:10.1161/CIRCULATIONAHA.113.003192 (2013).

S5 Wilde, E. *et al.* Tail-Cuff Technique and Its Influence on Central Blood Pressure in the Mouse. *J Am Heart Assoc* **6**, doi:10.1161/JAHA.116.005204 (2017).

S6 Fu, H. *et al.* Matrix metalloproteinase-7 protects against acute kidney injury by priming renal tubules for survival and regeneration. *Kidney Int* **95**, 1167-1180, doi:10.1016/j.kint.2018.11.043 (2019).

S7 Zhou, D. *et al.* Tubule-specific ablation of endogenous beta-catenin aggravates acute kidney injury in mice. *Kidney Int* **82**, 537-547, doi:10.1038/ki.2012.173 (2012).

S8 Zhou, D., Tan, R. J., Lin, L., Zhou, L. & Liu, Y. Activation of hepatocyte growth factor receptor, c-met, in renal tubules is required for renoprotection after acute kidney injury. *Kidney Int* **84**, 509-520, doi:10.1038/ki.2013.102 (2013).

S9 Gui, Y. *et al.* Fibroblast mTOR/PPARgamma/HGF axis protects against tubular cell death and acute kidney injury. *Cell Death Differ* **26**, 2774-2789, doi:10.1038/s41418-019-0336-3 (2019).

S10 Fu, H. *et al.* The hepatocyte growth factor/c-met pathway is a key determinant of the fibrotic kidney local microenvironment. *iScience* **24**, 103112, doi:10.1016/j.isci.2021.103112 (2021).

S11 Lin, Y. H. *et al.* Global Proteome and Phosphoproteome Characterization of Sepsis-induced Kidney Injury. *Mol Cell Proteomics* **19**, 2030-2047, doi:10.1074/mcp.RA120.002235 (2020).

S12 Lin, Y. H. *et al.* Self-Assembled STrap for Global Proteomics and Salivary Biomarker Discovery. *J Proteome Res* **18**, 1907-1915, doi:10.1021/acs.jproteome.9b00037 (2019).

S13 Yu, Y. & Pieper, R. Using Proteomics to Identify Inflammation During Urinary Tract Infection. *Methods Mol Biol* **2021**, 259-272, doi:10.1007/978-1-4939-9601-8_22 (2019).

S14 Yu, Y. & Pieper, R. Urinary pellet sample preparation for shotgun proteomic analysis of microbial infection and host-pathogen interactions. *Methods Mol. Biol.* **1295**, 65-74, doi:10.1007/978-1-4939-2550-6_6 (2015).

S15 Doncheva, N. T., Morris, J. H., Gorodkin, J. & Jensen, L. J. Cytoscape StringApp: Network Analysis and Visualization of Proteomics Data. *J Proteome Res* **18**, 623-632, doi:10.1021/acs.jproteome.8b00702 (2019).

S16 Gui, Y. *et al.* Calponin 2 harnesses metabolic reprogramming to determine kidney fibrosis. *Mol Metab* **71**, 101712, doi:10.1016/j.molmet.2023.101712 (2023).

S17 Chen, R. & Weng, Z. Docking unbound proteins using shape complementarity, desolvation, and electrostatics. *Proteins* **47**, 281-294, doi:10.1002/prot.10092 (2002).

S18 Li, L., Chen, R. & Weng, Z. RDOCK: refinement of rigid-body protein docking predictions. *Proteins* **53**, 693-707, doi:10.1002/prot.10460 (2003).

S19 Chen, R., Mintseris, J., Janin, J. & Weng, Z. A protein-protein docking benchmark. *Proteins* **52**, 88-91, doi:10.1002/prot.10390 (2003).

S20 Fu, H. *et al.* Tenascin-C Is a Major Component of the Fibrogenic Niche in Kidney Fibrosis. *J Am Soc Nephrol* **28**, 785-801, doi:10.1681/ASN.2016020165 (2017).

S21 Leman, J. K. *et al.* Macromolecular modeling and design in Rosetta: recent methods and frameworks. *Nat Methods* **17**, 665-680, doi:10.1038/s41592-020-0848-2 (2020).

S22 Maier, J. A. *et al.* ff14SB: Improving the Accuracy of Protein Side Chain and Backbone Parameters from ff99SB. *J Chem Theory Comput* **11**, 3696-3713, doi:10.1021/acs.jctc.5b00255 (2015).

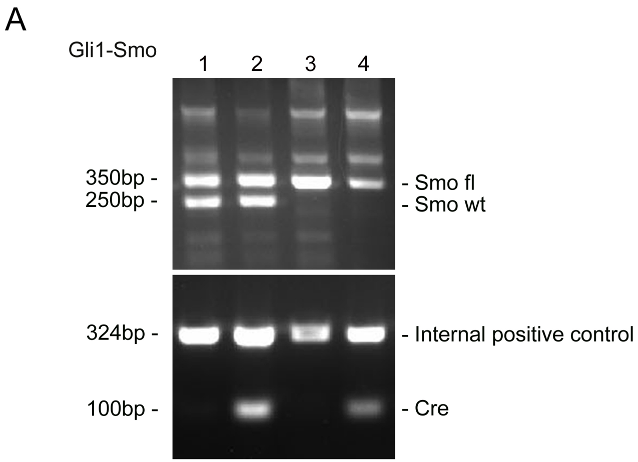

Supplementary Figure S1: (A) Genotyping of the mice by PCR analysis of genomic DNA. Lane 1 and 2 denote heterozygous control. Lane 3 shows the genotyping of the control mice used in this study (genotype: $Smo^{fl/fl}$), whereas lane 4 denotes the genotyping of the Gli1+ fibroblast-specific Smo knockout mice (genotype: $Smo^{fl/fl}$, Cre), designated as Gli1-Smo-/-. (B) Genotyping of the mice by PCR analysis of genomic DNA. Lane 1 shows the genotyping of the Pdgfrβ+ fibroblast-specific Smo knockout mice (genotype: $Smo^{fl/fl}$, Cre), designated as Pdgfrβ-Smo-/-, whereas lane 2 denotes the genotyping of the control mice used in this study (genotype: $Smo^{fl/fl}$). Lane 3 and 4 denote heterozygous control.

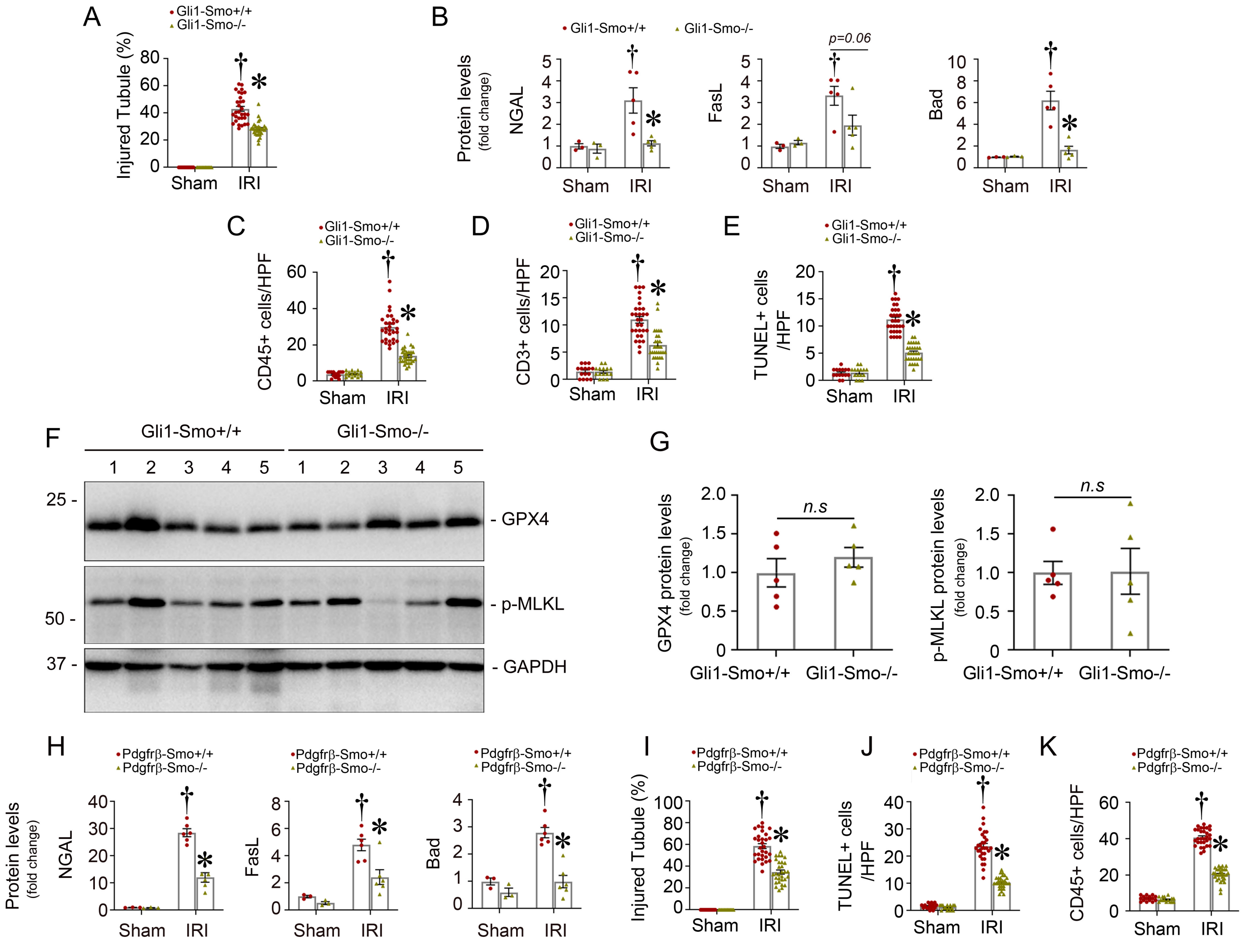

Supplementary Figure S2: Loss of Smo in fibroblasts reduced inflammatory cells infiltration and cell apoptosis but had little affects on cell ferroptosis and necroptosis after AKI. (A) Quantitative data for the kidney morphological changes between Gli1-Smo+/+ and Gli1-Smo-/- mice at 1 day after ischemic AKI. † $P < 0.05$ versus sham control, * $P < 0.05$ versus Gli1-Smo+/+ IRI mice (Sham, n=3; IRI, n = 6. 5 random images were selected per mouse, each dot represents the score of the according image). (B) Quantitative data for the protein expression of NGAL, FasL, and Bad in Gli1-Smo+/+ and Gli1-Smo-/- mice at 1 day after ischemic AKI. † $P < 0.05$ versus sham control, * $P < 0.05$ Gli1-Smo+/+ IRI mice (Sham, n = 3; IRI, n = 5). (C-E) Quantitative data for the kidney CD45 staing (C), CD3 staining (D), and TUNEL staining (E) between Gli1-Smo+/+ and Gli1-Smo-/- mice at 1 day after ischemic AKI. † $P < 0.05$ versus sham control, * $P < 0.05$ versus Gli1-Smo+/+ IRI mice (Sham, n=3; IRI, n = 6. 5 random images were selected per mouse, each dot represents the score of the according image).(F, G) Western blot assay demonstrated the changes of GPX4 and phosphorylated MLKL in Gli1-Smo+/+ and Gli1-Smo-/- diseased kidneys at 1 day after ischemic AKI (F). Quantitative data was presented in G (n = 5, each dot represents one mouse. n.s., non-significant). (H) Quantitative data for the protein expression of NGAL, FasL, and Bad in Pdgfrβ-Smo+/+ and Pdgfrβ-Smo-/- mice at 1 day after ischemic AKI. † $P < 0.05$ versus sham control, * $P < 0.05$ Pdgfrβ-Smo+/+ IRI mice (Sham, n = 3; IRI, n = 6). (I-K) Quantitative data for the kidney morphological changes (H), TUNEL staining (I), and CD45 staing (J) between Pdgfrβ-Smo+/+ and Pdgfrβ-Smo-/- mice at 1 day after ischemic AKI. † $P < 0.05$ versus sham control, * $P < 0.05$ versus Pdgfrβ-Smo+/+(Sham, n=3; IRI, n = 6. 5 random images were selected per mouse, each dot represents the score of the according image). Graphs are presented as mean ± SEM. Differences between groups were analyzed using unpaired t tests or one-way ANOVA followed by the Student-Newman-Keuls test.

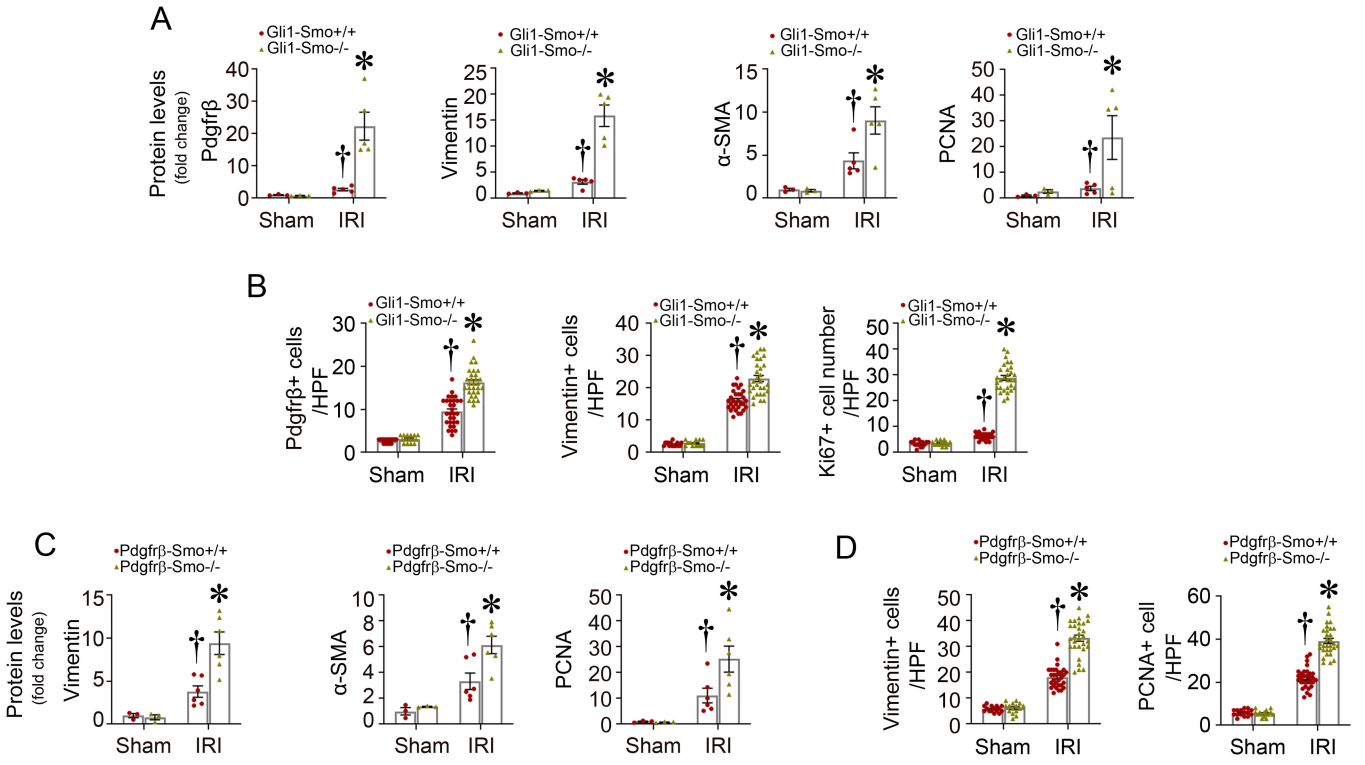

Supplementary Figure S3: Loss of Smo in fibroblasts enhances the activities of perivascular mesenchymal cells. (A) Quantitative data for PDGFR-β, vimentin, α-SMA, and PCNA proteins in the kidneys from Gli1-Smo+/+ and Gli1-Smo-/- mice at 1 day after ischemic AKI. Each dot represents one mouse. † *P* < 0.05 versus sham control, * *P* < 0.05 versus Gli1-Smo+/+ IRI mice. (Sham, n = 3; IRI, n = 5). (B) Quantitative data of PDGFR-β, vimentin, and Ki67 positive cell numbers *per* high-power field (HPF) in the kidneys from Gli1-Smo+/+ and Gli1-Smo-/- mice at 1 day after ischemic AKI. † *P* < 0.05 versus sham control, * *P* < 0.05 versus Gli1-Smo+/+ IRI mice. (Sham, n = 3; IRI, n = 6, 5 random images were selected *per* mouse, each dot represents the score of the according image). (C) Quantitative data for vimentin, α-SMA, and PCNA proteins in the kidneys from Pdgfrβ-Smo+/+ and Pdgfrβ-Smo-/- mice at 1 day after ischemic AKI. † *P* < 0.05 versus sham control, * *P* < 0.05 versus Pdgfrβ-Smo+/+ IRI mice. (Sham, n = 3; IRI, n = 6). Each dot represents one mouse. (D) Quantitative data of vimentin and Ki67 positive cell numbers *per* high-power field (HPF) in the kidneys from Pdgfrβ-Smo+/+ and Pdgfrβ-Smo-/- mice at 1 day after ischemic AKI. † *P* < 0.05 versus sham control, * *P* < 0.05 versus Pdgfrβ-Smo+/+ IRI mice. (Sham, n = 3; IRI, n = 6. 5 random images were selected *per* mouse, each dot represents the score of the according image). Graphs are presented as mean ± SEM. Differences between groups were analyzed using one-way ANOVA followed by the Student-Newman-Keuls test.

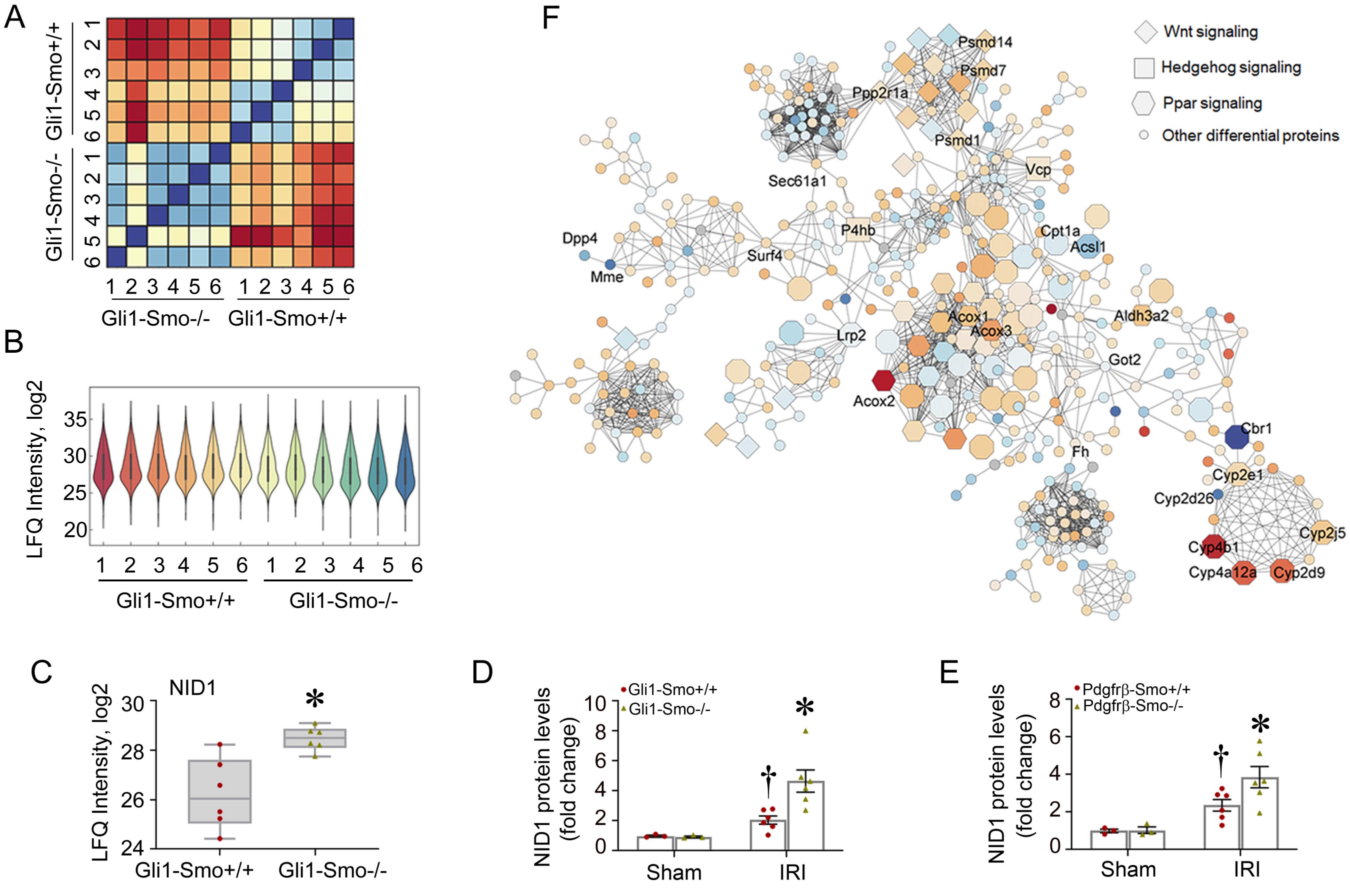

Supplementary Figure S4: Proteomics reveals altered signaling pathways after loss of Smo in fibroblast in AKI. (A) Correlation of the kidney proteome profiles between different groups of mice. Color scale represents R^2 values. (B) Violin plot of ANOVA significant proteins (Permutation FDR 0.05) among the Gli1-Smo+/+ and Gli1-Smo-/- mice. LFQ intensity of represented proteins was z-scored and plotted according to the color bar. (C) The intensity plot shows NID1 level changes between Gli1-Smo+/+ and Gli1-Smo-/- mice at 1 day after ischemic AKI. Box plots with median, min, max, and interquartile range. * $P < 0.05$ (n = 6). (D, E) Quantitative data of NID1 protein expression in Gli1-Smo+/+ and Gli1-Smo-/- or Pdgfrβ-Smo+/+ and Pdgfrβ-Smo-/- kidneys. † $P < 0.05$ versus sham control, * $P < 0.05$ versus Gli1-Smo+/+ or Pdgfrβ-Smo+/+ IRI mice (Sham, n = 3; IRI, n = 6). (F) Interaction network of the significantly changed signaling pathways, including Wnt, Hedgehog, and PPAR signaling after AKI in Gli1-Smo+/+ and Gli1-Smo-/- kidneys. The network and enrichment results were derived from String App in Cytoscape software. Graphs are presented as mean ± SEM. Differences between groups were analyzed using unpaired t tests or one-way ANOVA followed by the Student-Newman-Keuls test.

A

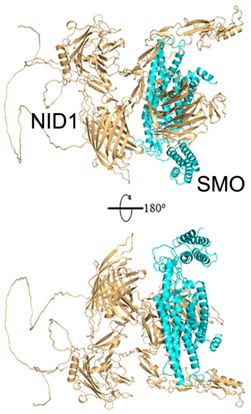

B

Name	ZDock Score	E_vdw1*	E_elec1*	E_vdw2*	E_elec2*	E_sol*	E_RDock*
**Pose 51**	19.54	-118.359	-0.320158	-153.525	-23.9267	-16.8	-38.334
**Pose 24**	17.66	-132.845	-4.26003	-130.678	-10.7906	-8.7	-18.4116
**Pose 553**	16.4	-30.5497	-4.75495	-196.764	0.670291	-7.6	-6.99674
**Pose 370**	17.68	-107.113	-2.03336	-106.271	6.19759	6.4	11.9778
**Pose 549**	16.96	-143.697	0.022321	-138.654	-3.92943	18	14.4635
**Pose 771**	15.96	-104.464	0.598036	-125.145	13.5521	8.5	20.6969
**Pose 985**	16.26	-148.794	-0.332786	-140.77	13.5788	9.8	22.0209
**Pose 823**	17.12	-105.273	2.44172	-117.466	7.63636	15.8	22.6727

* E_vdw1: van der Waals energy after first minimization with ionic residues in neutral state; E_vdw2: van der Waals energy after second minimization with ionic residues in charged state; E_elec1: Electrostatic energy after first minimization with ionic residues in neutral state; E_elec2: Electrostatic energy after second minimization with ionic residues in charged state; E_sol: Desolvation energy based on the Atomic Contact Energy.

E_RDock = E_sol + β*E_elec2. β refers to a scale factor that measures the contribution of desolvation energy and electrostatic energy to the total RDock energy.

C

NID1 Residue	SMO Residue	Interaction Constituents	Distance (Å)	Type
NID1:HIS724	SMO:TYR417	NID1:HIS724:HN - SMO:TYR417:OH	2.0211	Conventional
NID1:ASN729	SMO:ALA406	NID1:ASN729:HN - SMO:ALA406:O	2.9932	Conventional
NID1:ARG1017	SMO:ASN260	NID1:ARG1017:HH12 - SMO:ASN260:OD1	2.508	Conventional
NID1:ARG1017	SMO:ASN260	NID1:ARG1017:HH22 - SMO:ASN260:OD1	2.5953	Conventional
NID1:THR1014	SMO:TRP256	SMO:TRP256:HE1 - NID1:THR1014:O	2.8794	Conventional
NID1:THR1014	SMO:ASN260	SMO:ASN260:HD22 - NID1:THR1014:OG1	2.9579	Conventional
NID1:PRO931	SMO:GLN351	SMO:GLN351:HE21 - NID1:PRO931:O	2.8284	Conventional
NID1:ALA932	SMO:LYS356	SMO:LYS356:HZ1 - NID1:ALA932:O	2.2328	Conventional
NID1:GLY930	SMO:LYS356	SMO:LYS356:HZ2 - NID1:GLY930:O	2.345	Conventional
NID1:VAL933	SMO:THR357	SMO:THR357:HG1 - NID1:VAL933:O	1.9602	Conventional
NID1:ASP680	SMO:ARG398	SMO:ARG398:HH21 - NID1:ASP680:OD1	2.0129	Conventional

Supplementary Figure S5. RDOCK algorithm identifies multiple binding sites of NID1 and Smo in the optimal conformation at pose 51. (A) Binding pose of Smo and NID1; (B) Protein-protein RDock Performance shows pose 51 is the optimal conformation of Smo-NID1 interaction; (C) Hydrogen bond interactions of NID1 and Smo in the optimal conformation at pose 51.

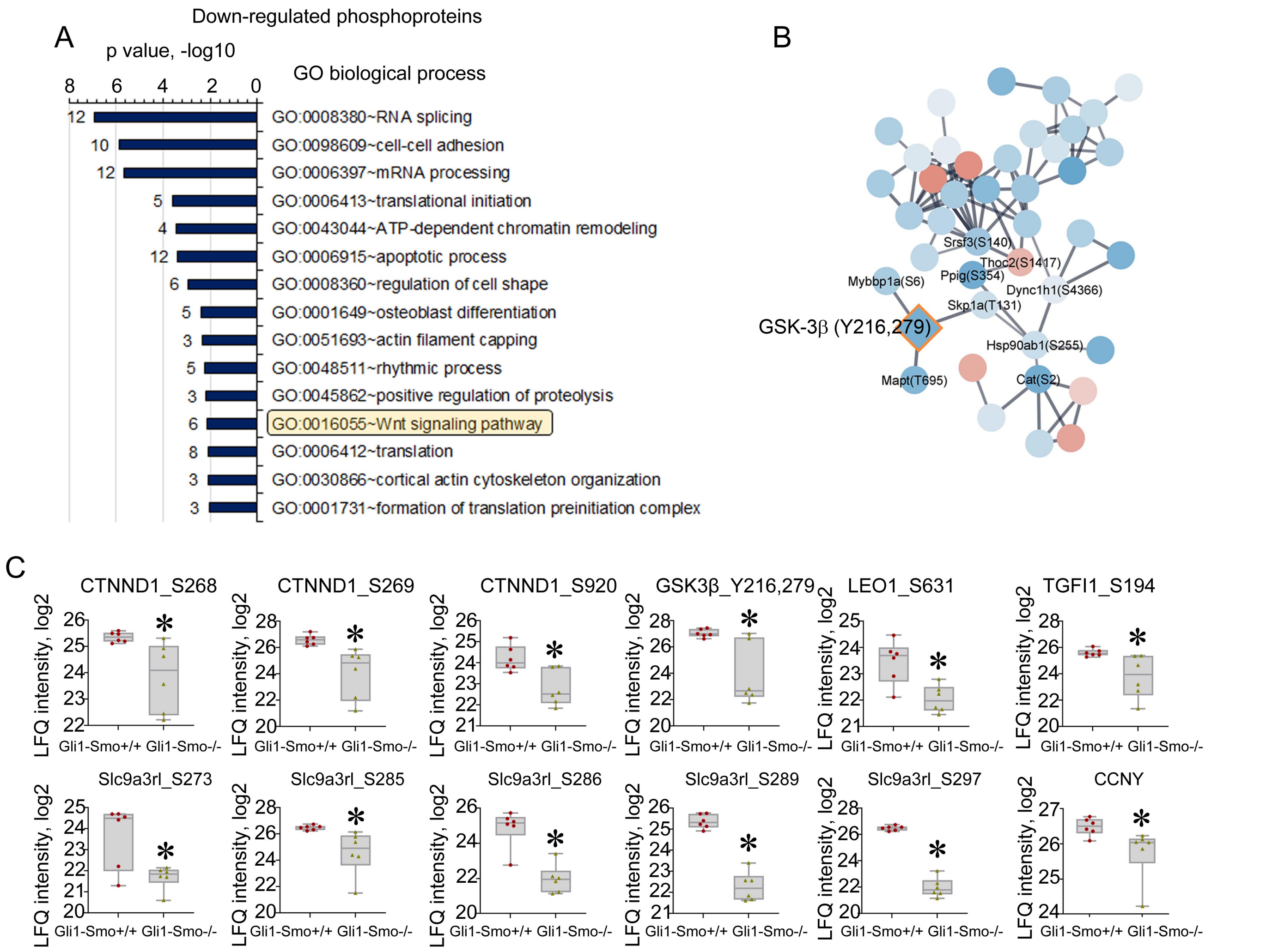

Supplementary Figure S6: Phosphoproteomics reveals Gli1+ fibroblast-derived Smo deletion remodels the Wnt signaling pathway. (A) Gene Ontology (GO) biological process analysis reveals key events (Wnt signaling is highlighted) between Gli1-Smo+/+ and Gli1-Smo-/- mice at 1 day after ischemic AKI. (B) Protein network derived from the STRING pathway database using the significant phosphoproteins as input. (C) Intensity plots of representative phosphosites of the components in Wnt signaling pathway and the changes in levels between Gli1-Smo+/+ and Gli1-Smo-/- mice at 1 day after ischemic AKI. Box plots with median, min, max, and interquartile range. n = 6. * $P < 0.05$. Each dot represents one mouse. Graphs are presented as mean ± SEM. Differences between groups were analyzed using unpaired t tests.

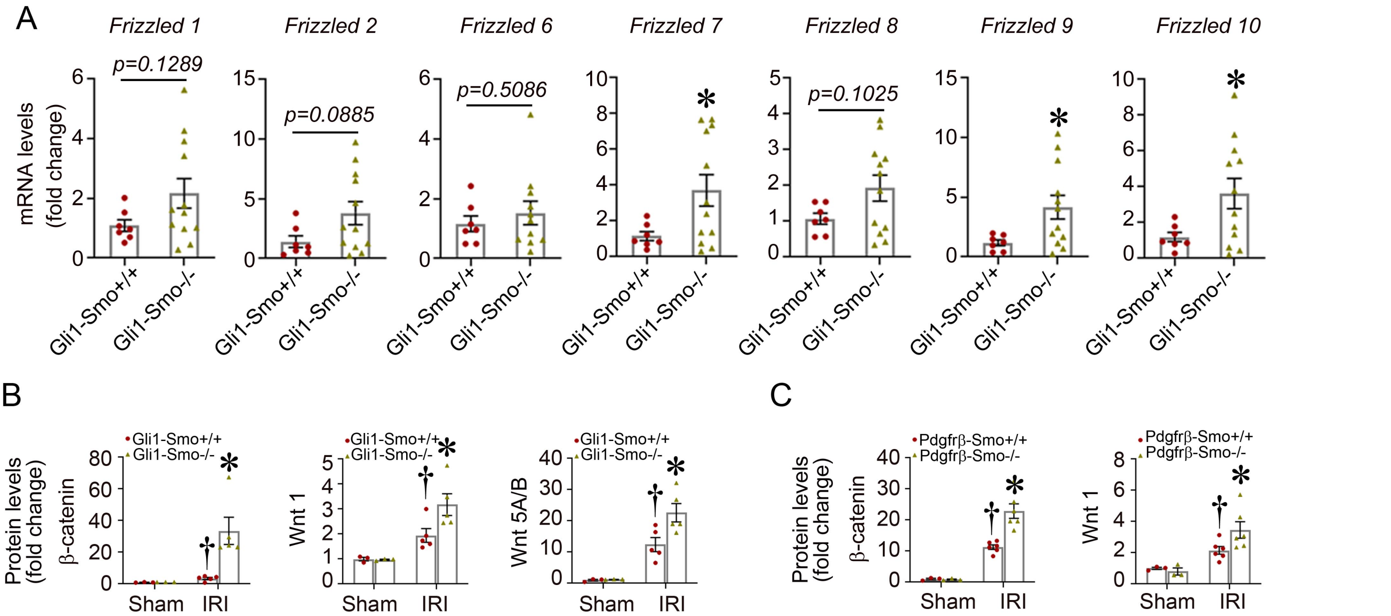

Supplementary Figure S7: Loss of Smo in fibroblasts activates the Wnt signaling pathway after AKI. (A) qRCR analysis revealing that 3 out of 10 Frizzled receptors were significantly upregulated in Gli1-Smo-/- mice at 1 day after AKI, compared to Gli1-Smo+/+. * $P < 0.05$ (n = 7-12). (B) Quantitative data of the levels of β-catenin, Wnt1, and Wnt5A/B proteins in the kidneys from Gli1-Smo+/+ and Gli1-Smo-/- mice at 1 day after ischemic AKI. † $P < 0.05$ versus sham control, * $P < 0.05$ versus Gli1-Smo+/+ IRI mice (Sham, n = 3; IRI, n = 5). Each dot represents one mouse. (C) Quantitative data of the levels of β-catenin and Wnt1 proteins in the kidneys from Pdgfrβ-Smo+/+ and Pdgfrβ-Smo-/- mice at 1 day after ischemic AKI. † $P < 0.05$ versus sham control, * $P < 0.05$ versus Pdgfrβ-Smo+/+ IRI mice (Sham, n = 3; IRI, n = 6). Each dot represents one mouse. Graphs are presented as mean ± SEM. Differences between groups were analyzed using unpaired t tests or one-way ANOVA followed by the Student-Newman-Keuls test.

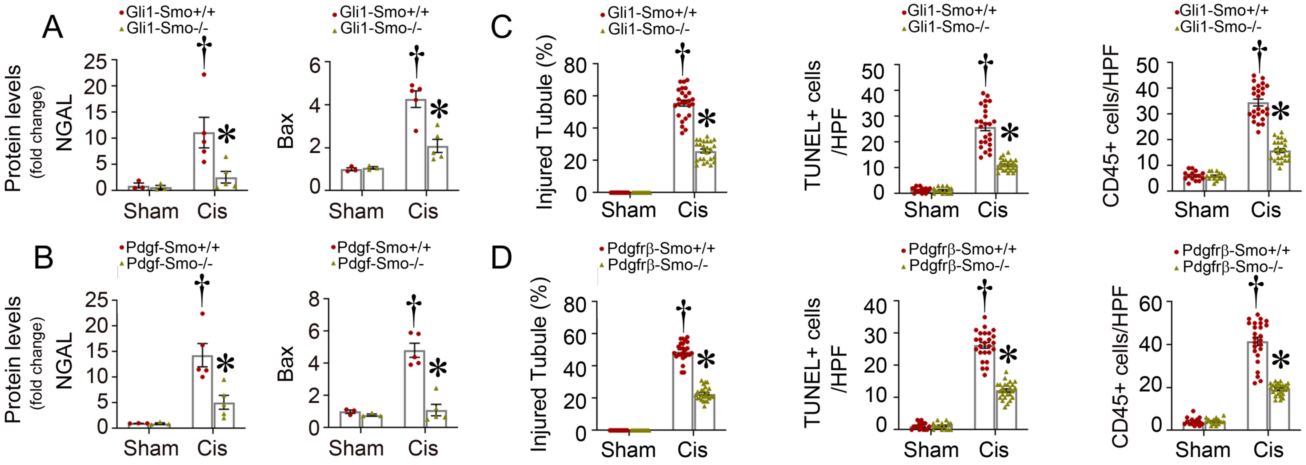

Supplementary Figure S8: Loss of Smo in fibroblasts reduced inflammatory cells infiltration and cell apoptosis after Cisplatin-induced AKI. (A, B) Quantitative data for NGAL and Bax proteins in the kidneys from Gli1-Smo+/+ and Gli1-Smo-/- mice (A) or Pdgfrβ-Smo+/+ and Pdgfrβ-Smo-/- mice (B) at 3 days after Cisplatin (Cis)-induced AKI. † *P* < 0.05 versus sham control, * *P* < 0.05 versus Gli1-Smo+/+ or Pdgfrβ-Smo+/+ IRI mice (Sham, n = 3, Cis, n = 5). Each dot represents one mouse. (C, D) Quantitative data for the kidney morphological changes, TUNEL assay, and CD45 staing between Gli1-Smo+/+ and Gli1-Smo-/- mice (C) or Pdgfrβ-Smo+/+ and Pdgfrβ-Smo-/- mice (D) at 3 days after Cisplatin-induced AKI. † *P* < 0.05 versus sham control, * *P* < 0.05 versus Gli1-Smo+/+ or Pdgfrβ-Smo+/+ IRI mice (Sham, n=3; Cis, n = 5. 5 random images were selected per mouse, each dot represents the score of the according image). HPF, high power field. Graphs are presented as mean ± SEM. Differences between groups were analyzed using one-way ANOVA followed by the Student-Newman-Keuls test.

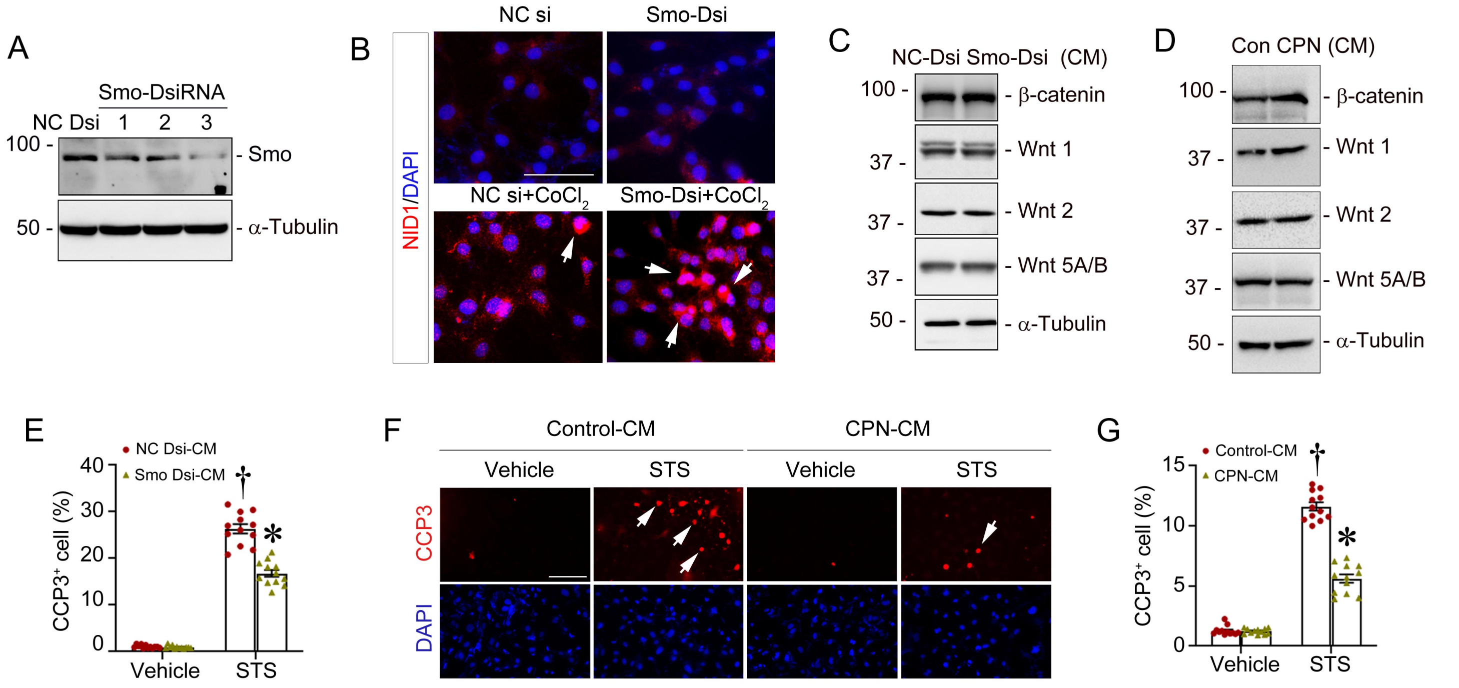

Supplementary Figure S9: Knockdown of Smo reduces tubular cell apoptosis in vitro. (A) Western blot analyses demonstrated the efficiency of knockdown Smo in normal rat kidney fibroblasts (NRK-49F) after transfected with dicer substrate siRNAs (Dsi). Numbers (1, 2, and 3) indicate each individual predesigned DsiRNA that are specific for Smo. (B) Immunofluorescence staining showed knockdown of Smo induced NID1 expression in cultured fibroblasts under hypoxia stress. Cells were co-stained with 4′,6-diamidino-2-phenylindole (DAPI) to visualize nuclei. Arrows indicate positive staining. Scale bar, 25 μm. (C, D) Representative western blots demonstrated that, under basal conditions, β-catenin, Wnt1, Wnt2, and Wnt5A/B protein levels had little changes after incubation with NID1-enriched conditioned medium (CM) collected from Smo-knockdown or cyclopamine (CPN)-treated fibroblasts. (E) Quantitative data for cleaved caspase 3 (CCP3) staining in tubular cells incubated with CM collected from Smo-knockdown fibroblasts under staurosporine (STS, 1 μM) stress. † $P < 0.05$ versus vehicles, * $P < 0.05$ versus NC Dsi-CM after STS (n = 3, 4 random images were selected per slide, each dot represents the score of the according image). (F, G) After stimulated with STS (1 μM) for 3 h, immunofluorescence staining showed fewer CCP3+ tubular cells incubated with NID1-enriched CM (F). Quantitative data are presented in G. Scale bar, 25 μm. Arrows indicate positive staining. † $P < 0.05$ versus vehicles, * $P < 0.05$ versus Control-CM after STS. (n = 3, 4 random images were selected per slide, each dot represents the score of the according image). Graphs are presented as means ± SEM. Differences between groups were analyzed using one-way ANOVA followed by the Student-Newman-Keuls test.

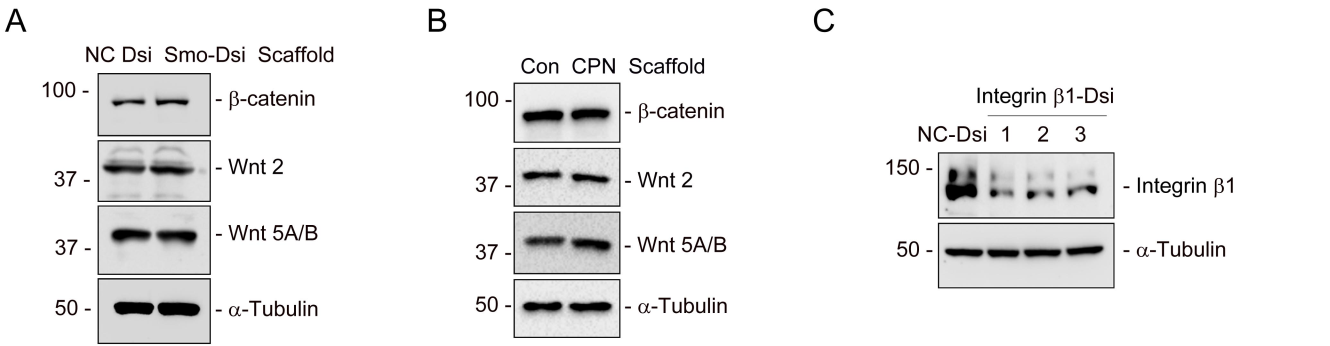

Supplementary Figure S10: Nidogen-1-enriched decellularized fibroblast matrix scaffold activates Wnt components *ex vivo*. (A, B) Western blots assay demonstrated that Wnt components β-catenin, Wnt2, and Wnt5A/B appeared unchanged in normal rat kidney proximal tubular cells (NRK-52E) seeded on NID1-enriched matrix scaffold under basal conditions. (C) Western blot analyses demonstrated the efficiency of knockdown Integrin-β1 in NRK-52E after transfected with dicer substrate siRNAs (Dsi). Numbers (1, 2, and 3) indicate each individual predesigned DsiRNA that are specific for integrin-β1.

A

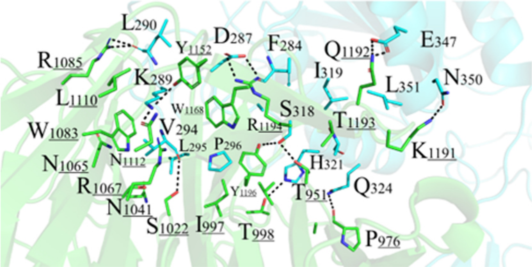

Binding interface

B

	Molecular Docking Energy (kcal/mol)
**Binding site**	-35.7712

C

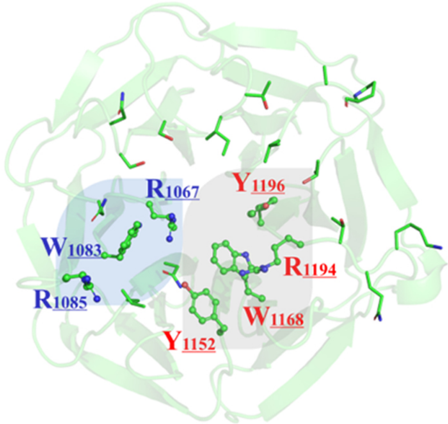

Supplementary Figure S11: Molecular docking analysis shows the binding patterns between NID1 and Integrin β1. (A) The selected binding site between NID1 and Integrin β1 proteins form a dense hydrogen bonding network system, which play key roles in the stable binding between two proteins. The molecular docking energy of the binding site is presented (B). (C) The selected amino acids on NID1 protein that have greater influences on the NID1-Integrin β1 interactions.

Supplementary Table S4. Nucleotide sequences of the primers used for qRT-PCR

Mouse gene	Primer Sequence 5’ to 3’	
	**Forward**	**Reverse**
Smo	CGGACTCGCAGGAGGAAGCG	GGGTACGGCTGGGCAACTCC
TNFα	CCCTCACACTCAGATCATCTTCT	CCCTCACACTCAGATCATCTTCT
MCP1	TTAAAAACCTGGATCGGAACCAA	GCATTAGCTTCAGATTTACGGGT
Rantes	GCTGCTTTGCCTACCTCTCC	TCGAGTGACAAACACGACTGC
FSP1	AGCTACTGACCAGGGAGCTG	TCATTGTCCCTGTTGCTGTC
Vimentin	AGAGAGAGGAAGCCGAAAGC	GCTCCTGGATCTCTTCATCG
α-SMA	GAGGCACCACTGAACCCTAA	CATCTCCAGAGTCCAGCACA
Wnt1	CGACTGATCCGACAGAACCC	AGCCTCGGTTGACGATCTTG
Wnt2	GCTCTTGACCTGGCTCACCC	CAGGCCAATGGCACGCAT
Wnt4	ATCTCTTCAGCAGGTGTGGC	TGTTGTCCGAGCATCCTGAC
Wnt5a	AGACCTCAGAGGGGATGGAC	TCTCCGTGCACTTCTTGCAT
Wnt5b	GTGCAGAGACCGGAGATGTT	ACTGGTGTTGGCACTCTCTG
Wnt7a	CGGACGCCATCATCGTCATA	CACTTTGAGCTCCTTCCCGA
Wnt7b	TCCTCTACGTGAAGCTCGGA	TCCCCGATCACAATGATGGC
Wnt9b	CAGGTGCTGAAGCTACGCTA	CTTCCATGTAGACCAGGTCCC
Wnt10a	GGTAAACTGAAGGCTTGCGG	AAGTATGGCCGGGTGTTCAG
Wnt10b	CAAGACCGGCTTAGAGCCAA	TCCATGTCGTGGTTACAGCC
Wnt11	ACTGTAAACAGCTGGAGGGC	CGATGGAGGAGCAGTTCCAG
Wnt16	TACGGCATGTGGTTCAGCAG	GCGGCAGTCCACAGACATTA
Frizzled 1	CGTCGGTTTCAAGGCTCCTC	GTGTCAATCCCTCAACTCGC
Frizzled 2	AAGAACTCTGCCCAACACCC	CACTCTTCAGCCCTGGTGTC
Frizzled 6	CAGAGTCAGAGTTGGGCAGG	AAGGAGGACCAGCACTGAGA
Frizzled 7	GGGGCGAGAGATGGTTTTGA	CGTCCTGCAAGTCTTAGCCA
Frizzled 8	GGCCATTCTACTTGGCCTGT	GAGTGCAGCCAGCTCTGTAA
Frizzled 9	GTGGGGACCCAGTAAAGCTC	TATCCTGGGACCCTCTGTGG
Frizzled 10	TGTGACAACCCAGGCAAGTT	CGATGGGTCGATGAGGAAGG
β-actin	CAGCTGAGAGGGAAATCGTG	CGTTGCCAATAGTGATGACC

Supplementary Table S5. The information of the applied primary and secondary antibodies

Name	Vendor	Category Number	Application
Smo	Santa Cruz Biotechnology, Dallas, TX	sc-166685	WB (in vitro), Co-IP
Smo	MyBiosource, San Diego, CA	MBS7051048	WB (Primary cells)
CD45	Cell signaling Technology, Danvers, MA	#70257	IHC
CD3	Abcam, Cambridge, MA	ab135372	IF
NGAL	Abcam, Cambridge, MA	ab63929	WB
FasL	Santa Cruz Biotechnology, Dallas, Texas	sc-19681	WB
Bad	Santa Cruz Biotechnology, Dallas, Texas	sc-8044	WB
PDGFR-β	Cell signaling Technology, Danvers, MA	#3169	WB, IF
Vimentin	Cell signaling Technology, Danvers, MA	#5741	WB, IHC
α-SMA	Abcam, Cambridge, MA	ab5694	WB
PCNA	Cell signaling Technology, Danvers, MA	#13110	WB, IHC
Ki67	Cell signaling Technology, Danvers, MA	#12202	IHC
NID1	Abcam, Cambridge, MA	ab254325	WB, IHC
NID1	Santa Cruz Biotechnology, Dallas, TX	sc-33706	Co-IP
β-catenin	Proteintech Group, Rosemont, IL	51067-2-AP	WB, IHC
Wnt 1	Proteintech Group, Rosemont, IL	27935-1-AP	WB, IHC
Wnt 4	Proteintech Group, Rosemont, IL	14371-1-AP	IHC
Wnt 5A/B	Proteintech Group, Rosemont, IL	55184-1-AP	WB, IHC
Integrin β1	Proteintech Group, Rosemont, IL	26918-1-AP	WB, IHC
Integrin β1	Santa Cruz Biotechnology, Dallas, TX	sc-374429	WB
Integrin α3	Proteintech Group, Rosemont, IL	21992-1-AP	WB
Integrin α6	Proteintech Group, Rosemont, IL	27189-1-AP	WB
Integrin αV	Proteintech Group, Rosemont, IL	27096-1-AP	WB
Integrin α8	Santa Cruz Biotechnology, Dallas, TX	sc-365798	WB
Integrin β6	Proteintech Group, Rosemont, IL	19695-1-AP	WB
Bax	Santa Cruz Biotechnology, Dallas, TX	sc-7480	WB
Wnt 2	Proteintech Group, Rosemont, IL	27214-1-AP	WB
Wnt 16	Proteintech Group, Rosemont, IL	28610-1-AP	WB
Cleaved Caspase-3	Cell signaling Technology, Danvers, MA	#9664	WB, IF
Caspase3	Cell signaling Technology, Danvers, MA	#9662	WB
P-MLKL	Abcam, Cambridge, MA	ab196436	WB
GPX4	Abcam, Cambridge, MA	ab125066	WB
α-Tubulin	Sigma, St. Louis, MO	T9026	WB
GAPDH	Santa Cruz Biotechnology, Dallas, Texas	sc-32233	WB
Anti-Mouse IgG	Abcam, Cambridge, MA	ab6789	WB
Anti-Rabbit IgG	Abcam, Cambridge, MA	ab6721	WB
Cy3 Anti-Rabbit	Jackson ImmunoResearch	711-165-152	IF
Alexa Fluor® 488 Anti-Rabbit	Jackson ImmunoResearch	711-545-152	IF
Biotin-Anti-Rabbit	Jackson ImmunoResearch	711-065-152	IHC

WB, western blot; IHC, Immunohistochemical staining; IF, Immunofluorescence staining; Co-IP, Co-immunoprecipitation
