# Supplementary material for: Fibroblast expression of transmembrane protein smoothened governs microenvironment characteristics after acute kidney injury
Source: J Clin Invest. 2024 May 7;134(13):e165836. doi: 10.1172/JCI165836 (PMC11213467; doi:10.1172/JCI165836)
Supplement: Unedited blot and gel images [file jci-134-165836-s094.pdf]

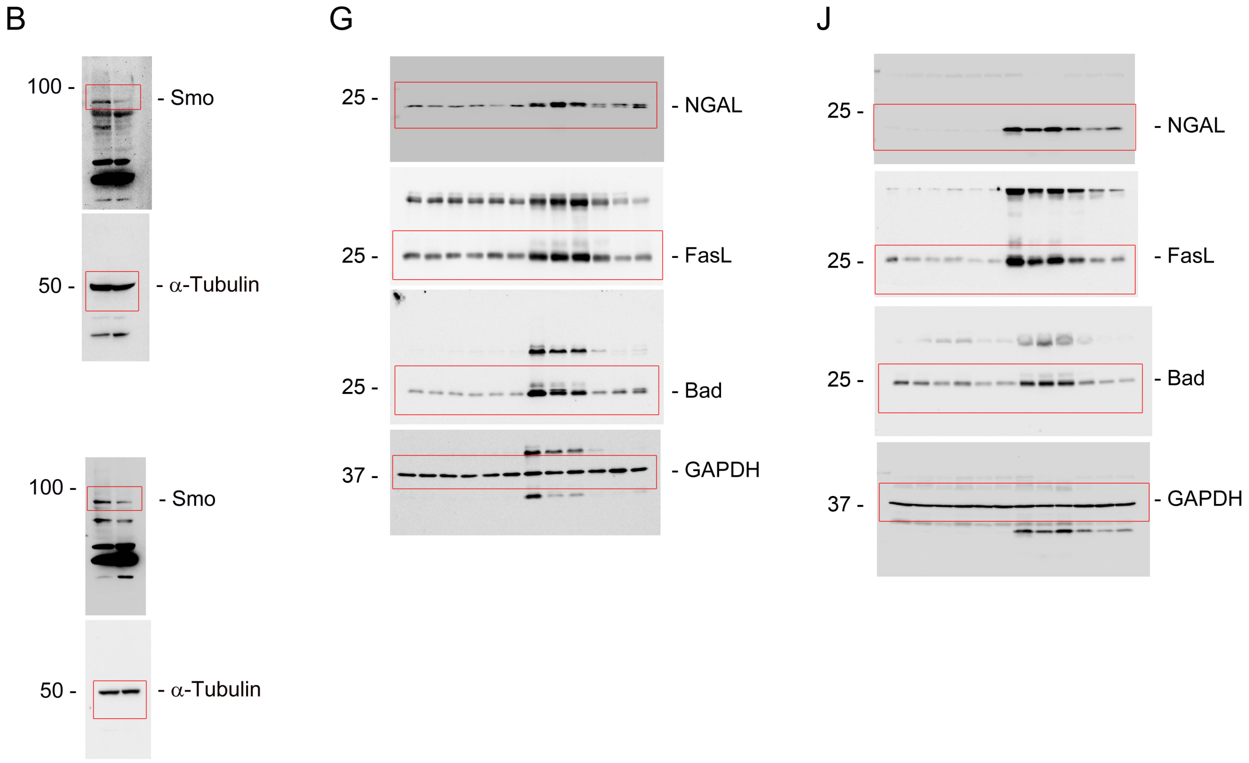
B
100 -
- Smo
50 -
- α-Tubulin
100 -
- Smo
50 -
- α-Tubulin
G
25 -
- NGAL
25 -
- FasL
25 -
- Bad
37 -
- GAPDH
J
25 -
- NGAL
25 -
- FasL
25 -
- Bad
37 -
- GAPDH

Figure 1

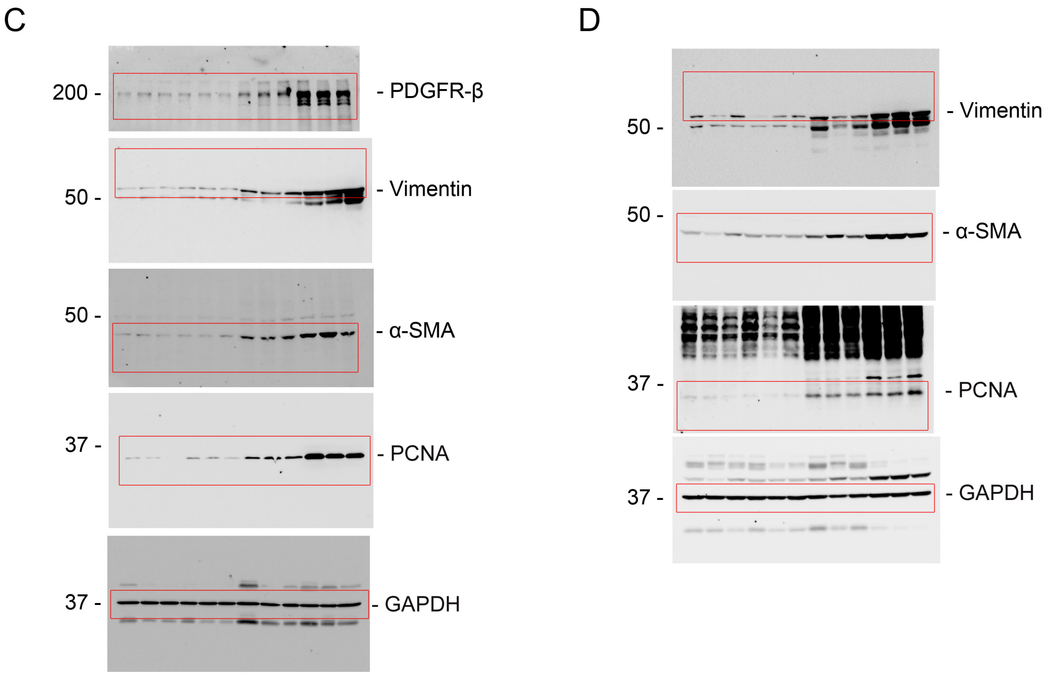
C
200 -
- PDGFR-β
50 -
- Vimentin
50 -
- α-SMA
37 -
- PCNA
37 -
- GAPDH
D
50 -
- Vimentin
50 -
- α-SMA
37 -
- PCNA
37 -
- GAPDH

Figure 2

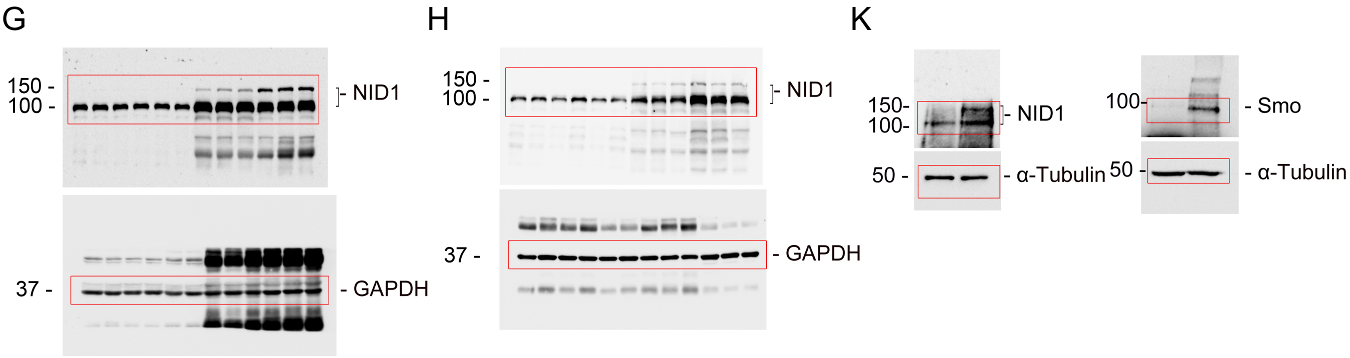
G
150 -
100 -
- NID1
37 -
- GAPDH
H
150 -
100 -
- NID1
37 -
- GAPDH
K
150-
100-
- NID1
50 -
- α-Tubulin
100-
- Smo
50 -
- α-Tubulin

Figure 3

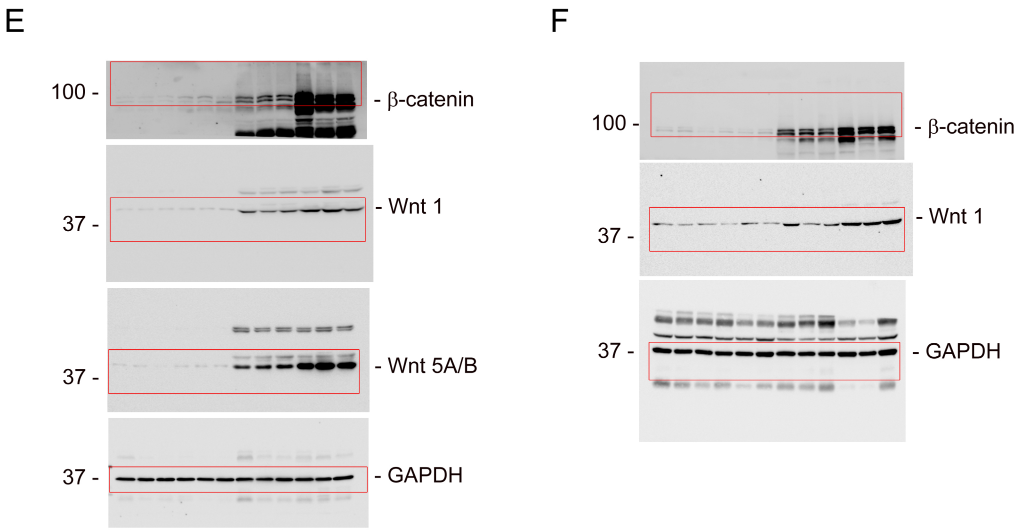
E
100 -
- β-catenin
- Wnt 1
37 -
37 -
- Wnt 5A/B
37 -
- GAPDH
F
100 -
- β-catenin
- Wnt 1
37 -
37 -
- GAPDH

Figure 4

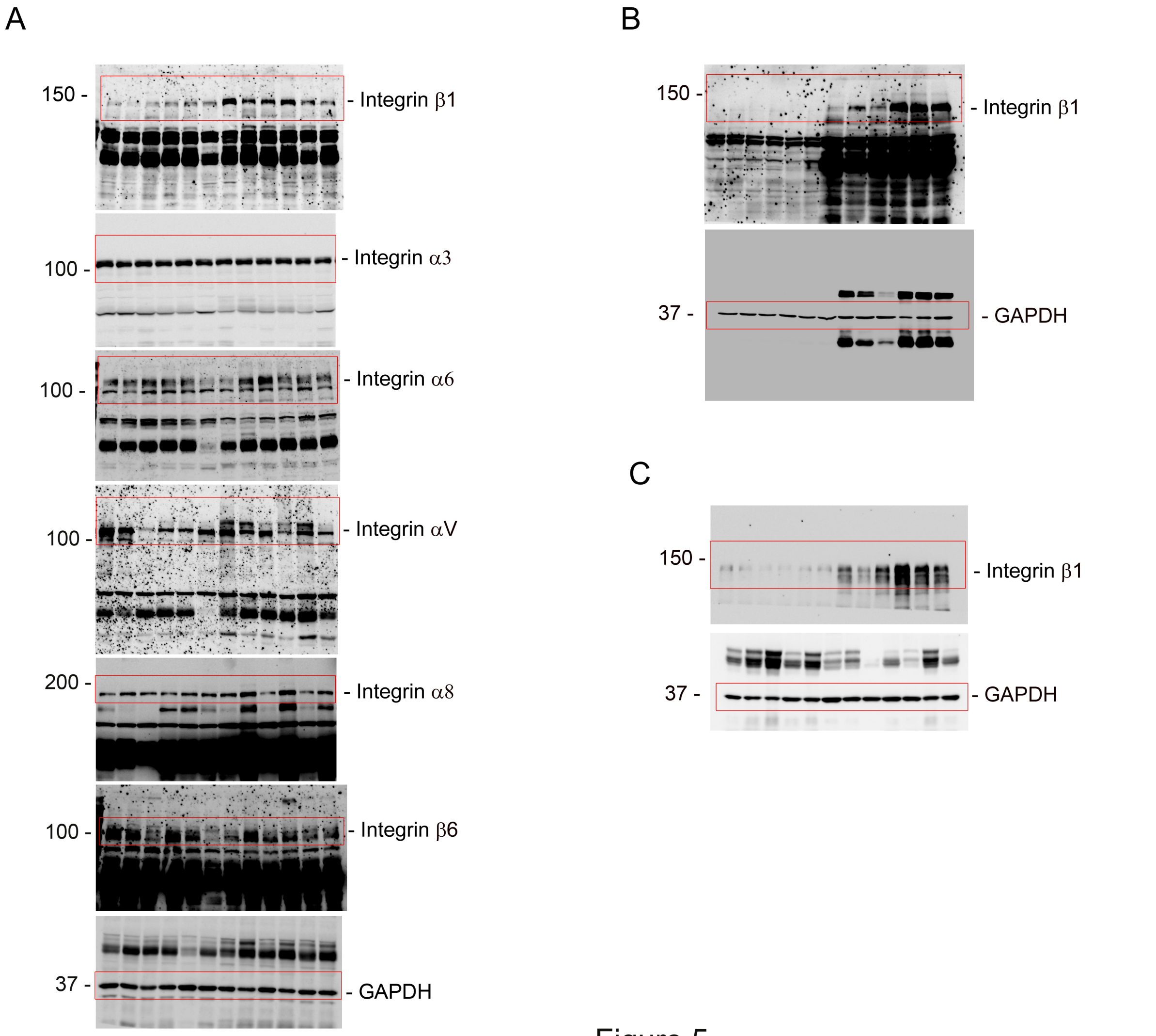
A
150 -
- Integrin β1
100 -
- Integrin α3
100 -
- Integrin α6
100 -
- Integrin αV
200 -
- Integrin α8
100 -
- Integrin β6
37 -
- GAPDH
B
150 -
- Integrin β1
37 -
- GAPDH
C
150 -
- Integrin β1
37 -
- GAPDH

Figure 5

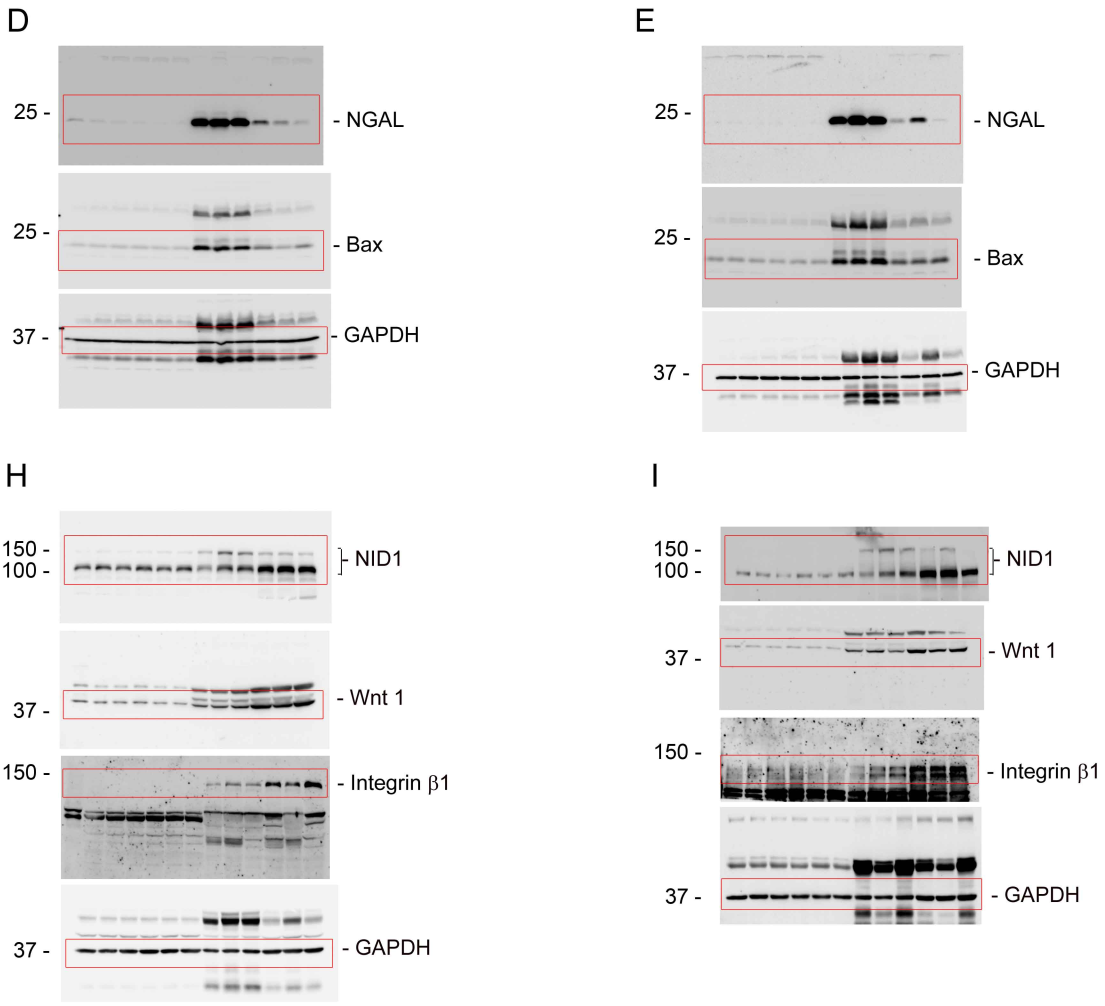
D
25 -
- NGAL
25 -
- Bax
37 -
- GAPDH
E
25 -
- NGAL
25 -
- Bax
37 -
- GAPDH
H
150 -
100 -
- NID1
37 -
- Wnt 1
150 -
- Integrin β1
37 -
- GAPDH
I
150 -
100 -
- NID1
37 -
- Wnt 1
150 -
- Integrin β1
37 -
- GAPDH

Figure 6

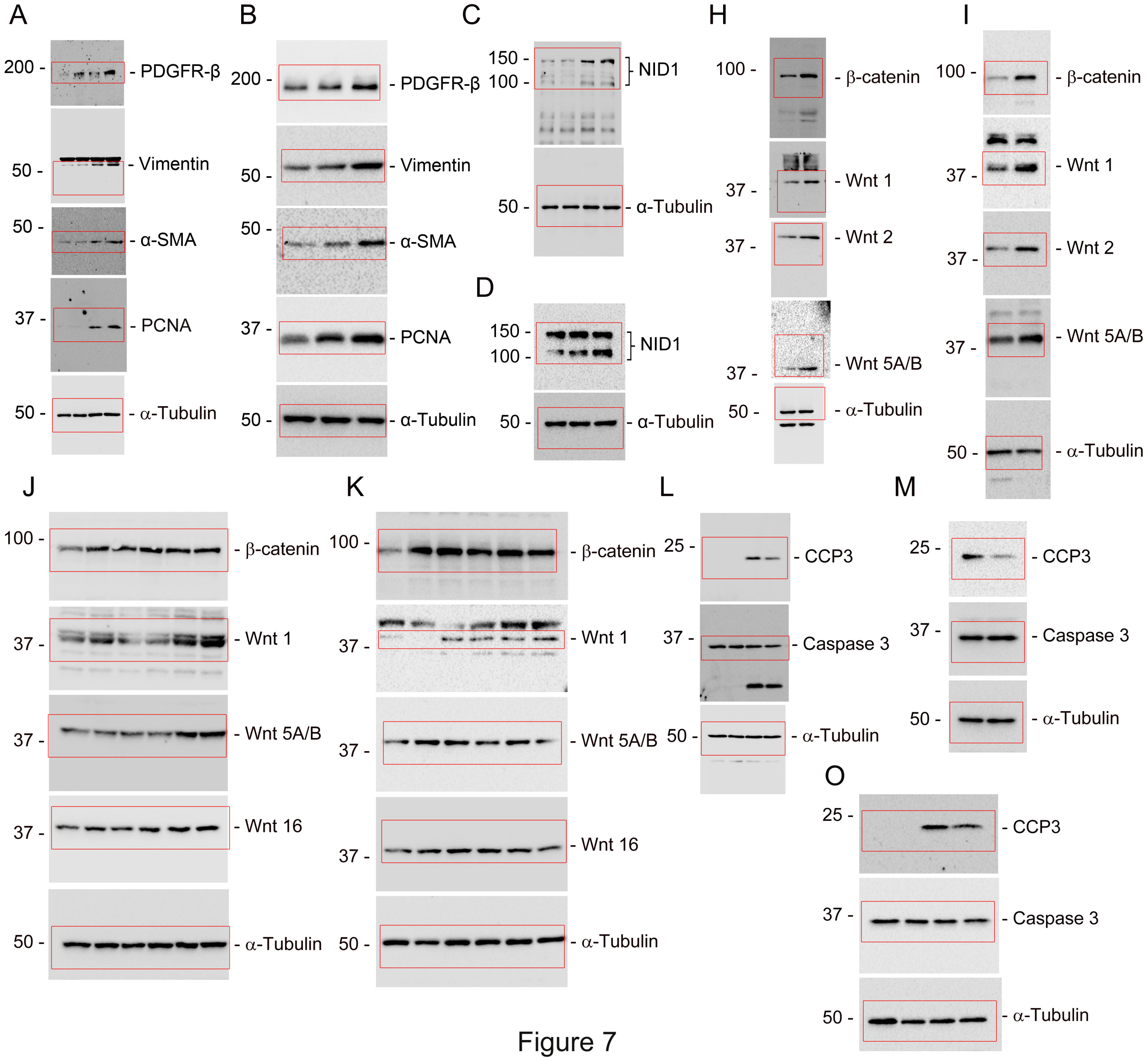
A
200 -
- PDGFR-β
50 -
- Vimentin
50 -
- α-SMA
37 -
- PCNA
50 -
- α-Tubulin
B
200 -
- PDGFR-β
50 -
- Vimentin
50 -
- α-SMA
37 -
- PCNA
50 -
- α-Tubulin
C
150 -
100 -
NID1
50 -
- α-Tubulin
D
150 -
100 -
NID1
50 -
- α-Tubulin
H
100 -
- β-catenin
37 -
- Wnt 1
37 -
- Wnt 2
37 -
- Wnt 5A/B
50 -
- α-Tubulin
I
100 -
- β-catenin
37 -
- Wnt 1
37 -
- Wnt 2
37 -
- Wnt 5A/B
50 -
- α-Tubulin
J
100 -
- β-catenin
37 -
- Wnt 1
37 -
- Wnt 5A/B
37 -
- Wnt 16
50 -
- α-Tubulin
K
100 -
- β-catenin
37 -
- Wnt 1
37 -
- Wnt 5A/B
37 -
- Wnt 16
50 -
- α-Tubulin
L
25 -
- CCP3
37 -
- Caspase 3
50 -
- α-Tubulin
M
25 -
- CCP3
37 -
- Caspase 3
50 -
- α-Tubulin
O
25 -
- CCP3
37 -
- Caspase 3
50 -
- α-Tubulin

Figure 7

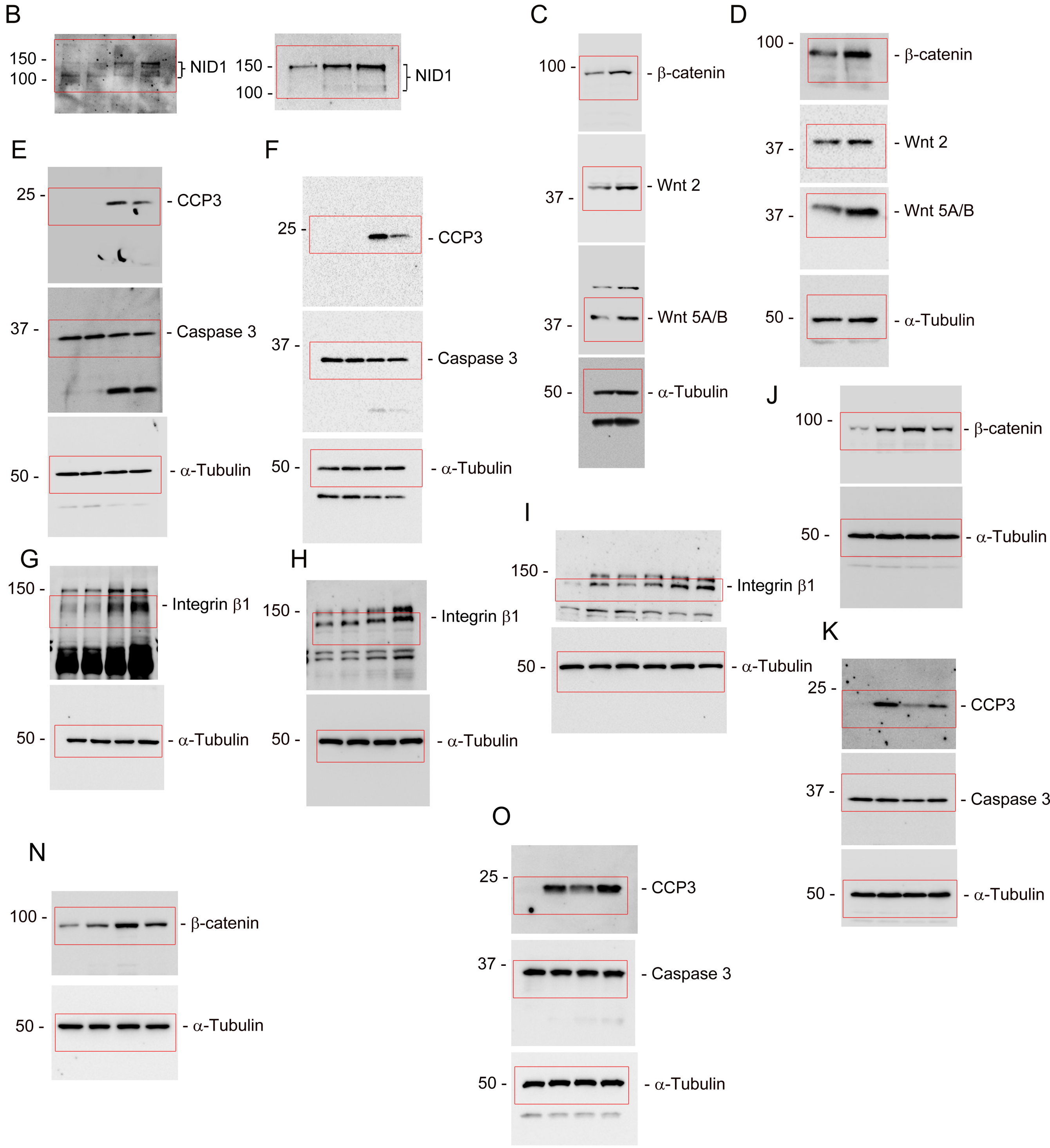
B
150 -
100 -
NID1
150 -
100 -
NID1
C
100 -
- β-catenin
37 -
- Wnt 2
37 -
- Wnt 5A/B
50 -
- α-Tubulin
D
100 -
- β-catenin
37 -
- Wnt 2
37 -
- Wnt 5A/B
50 -
- α-Tubulin
E
25 -
- CCP3
37 -
- Caspase 3
50 -
- α-Tubulin
F
25 -
- CCP3
37 -
- Caspase 3
50 -
- α-Tubulin
J
100 -
- β-catenin
50 -
- α-Tubulin
I
150 -
- Integrin β1
50 -
- α-Tubulin
G
150 -
- Integrin β1
50 -
- α-Tubulin
H
150 -
- Integrin β1
50 -
- α-Tubulin
K
25 -
- CCP3
37 -
- Caspase 3
50 -
- α-Tubulin
N
100 -
- β-catenin
50 -
- α-Tubulin
O
25 -
- CCP3
37 -
- Caspase 3
50 -
- α-Tubulin

Figure 8

F

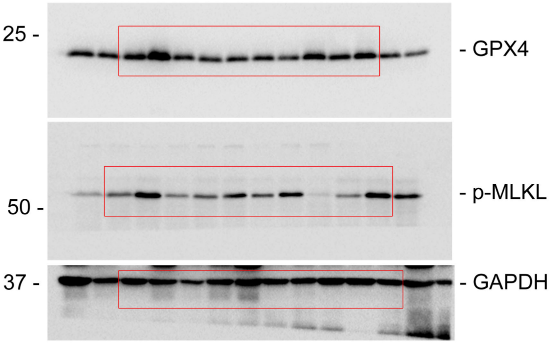

Supplementary Figure S2

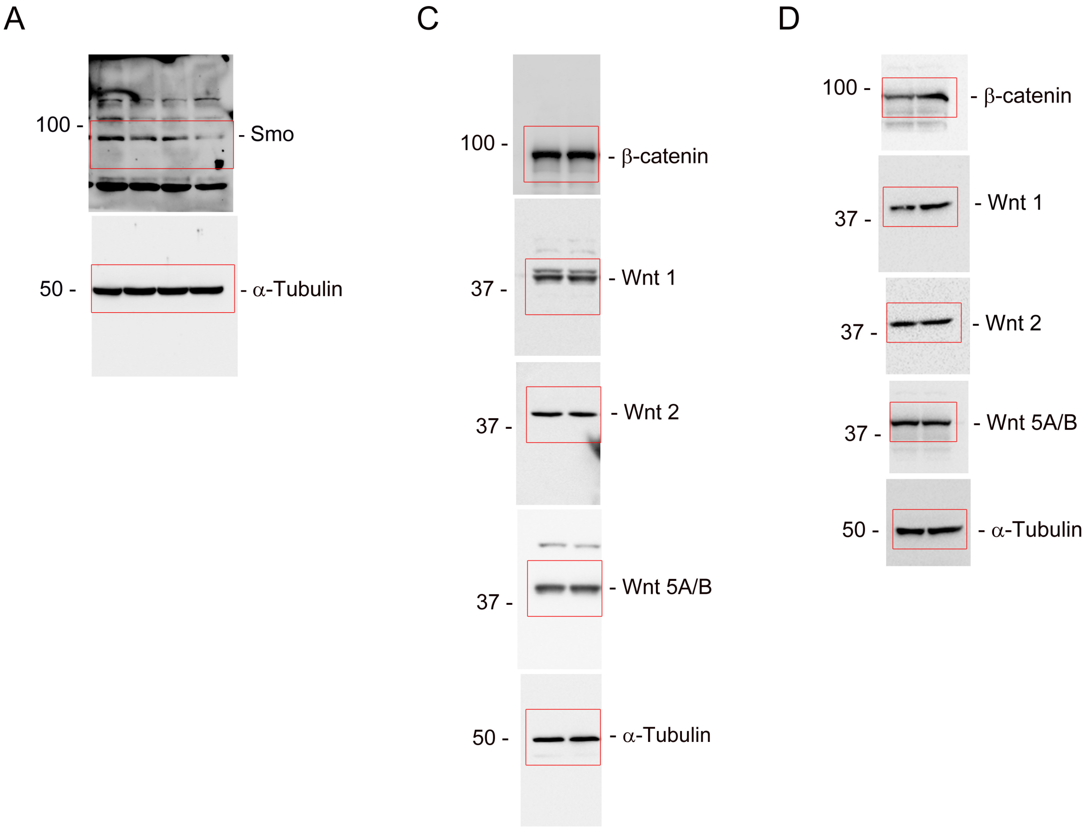

Supplementary Figure S9

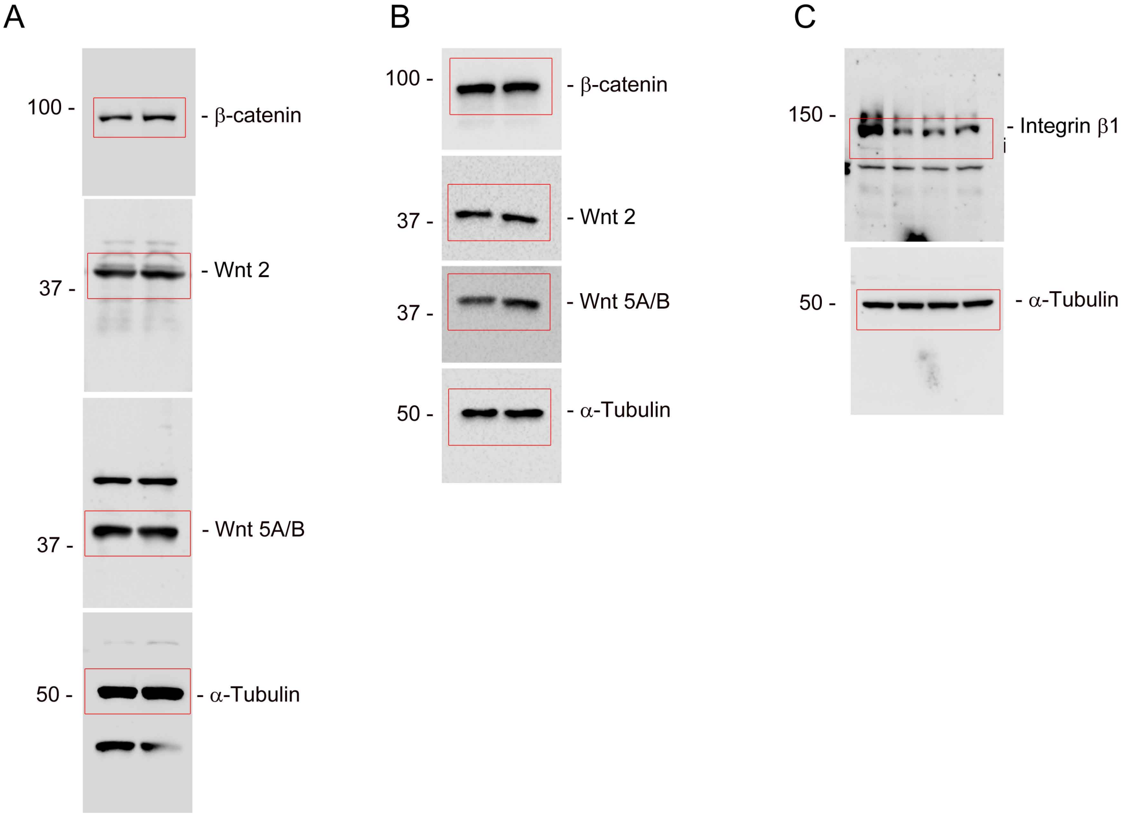

Supplementary Figure S10
